# Therapeutic potential of *Abelmoschus manihot*: mechanisms of action and clinical use in traditional Chinese medicine formulas

**DOI:** 10.3389/fphar.2025.1709530

**Published:** 2025-12-10

**Authors:** Chu Xue, Haitao Ge, Yaru Liu, Yan Zhao, Wenjie Huang, Zhaowen Lu, Qiuhui Ye, Xiaoli Chen, Zhengyu Cao

**Affiliations:** 1 State Key Laboratory of Natural Medicines and Jiangsu Provincial Key Laboratory for TCM Evaluation and Translational Development, School of Traditional Chinese Pharmacy, China Pharmaceutical University, Nanjing, Jiangsu, China; 2 Jiangsu Suzhong Pharmaceutical Group Co., Ltd., Taizhou, Jiangsu, China; 3 The Department of Ultrasound of Sir Run Run Shaw Hospital Affiliated to Nanjing Medical University, Nanjing, Jiangsu, China

**Keywords:** *Abelmoschus manihot*, Huangkui Capsule, total flavonoids of *Abelmoschus Manihot*, Jiahua Tablets, Chuangling Liquid, Huangkui Lianchang Decoction

## Abstract

*Abelmoschus manihot* (L.) Medicus (AM), commonly known as Huangshukui in China, is a traditional medicinal herb. Its flowers serve as the primary active component of Huangkui Capsule (HKC), which has demonstrated therapeutic potential in various conditions such as chronic kidney disease (CKD), inflammatory bowel disease (IBD), ischemic cardiac/cerebral injuries, hepatic injury, and diabetes mellitus. In order to reveal that AM has extensive clinical applications and significant development value, this paper collates the pharmacological effects of AM and the clinical data of traditional Chinese medicine (TCM) formulations containing AM. This review aims to systematically summarize the pharmacological effects and clinical applications of AM, with a focus on its underlying mechanisms—including immunomodulation, antifibrotic activity, metabolic regulation, intestinal flora modulation, organ protection, antioxidant effects, and analgesia. Although most clinical data currently center on HKC, this article also examines other TCM formulations containing AM, such as Jiahua Tablets, Chuangling Liquid, Huangkui Lianchang Decoction, Huangkui Siwu Formula, Yu Kui Qing, Qikui Granules, Huangshukui paste, and Er Huang Ointment. By consolidating current evidence on the pharmacology and clinical use of AM, this review highlights its broad therapeutic potential and promote further research and development of AM-based treatments.

## 1 Introduction

*Abelmoschus manihot* (L.) Medicus (AM), known as Huangshukui in Chinese, is a versatile herbaceous plant of the Malvaceae family (Abelmoschus genus) with significant medicinal and edible value (Hong et al., 2021). Its therapeutic importance is well-established both in history and in modern medicine. Ancient Chinese medical books, such as *Jiayou Materia Medica* and the *Compendium of Materia Medica*, document AM’s use for treating conditions like carbuncles, toxic swellings, and scalds (Liu et al., 2023; Xue et al., 2023). The 2020 edition of the *Pharmacopoeia of the People’s Republic of China* (*Chinese Pharmacopoeia*) formally recognizes the AM flower for clearance of dampness and heat from the body, diminishing swelling, and eliminating toxins (Luan et al., 2020). Beyond China, AM (also known as Aibika or Sunset Muskmallow) is widely used in countries including India, Nepal, Papua New Guinea, Vanuatu, Fiji, and New Caledonia for various medicinal purposes. In these regions, its applications range from using crushed seeds to relieve pain and foot spasms, as well as to manage lactation or menorrhagia, to the practice of applying root juice for sprains (Todarwal et al., 2011; Zhong et al., 2024). 

Although the dried flower is the primary part used in medicine, other plant components—such as the seeds, roots, stems, and leaves—are also utilized to address conditions like indigestion, poor appetite, and traumatic injuries (Yin et al., 2021). Phytochemical studies have identified diverse constituents in AM, such as flavonoids, polysaccharides, steroids, volatile oils, and amino acids (Liu et al., 2016; Guo et al., 2021; Xue et al., 2023). Modern pharmacological research validates and expands upon traditional uses, revealing that AM possesses various bioactivities, including antimicrobial, antioxidant, analgesic, and anti-inflammatory properties. These mechanisms contribute to the treatment of various ailments, such as the amelioration of inflammatory bowel disease (IBD), restoration of heart and brain damage, improvement of renal and liver function, and regulation of intestinal flora and glucose/lipid metabolism (Ding et al., 2013; Zhang et al., 2017; Zhu et al., 2018; Luan et al., 2020; Gao et al., 2022; Zhou et al., 2022; Wei et al., 2023; Miao et al., 2024b; Song et al., 2024; Xu et al., 2024; Zhang et al., 2024; Wen and Chen, 2017; Zhou and Chen, 2016).

The most widespread application of AM in clinical practice is Huangkui Capsule (HKC), a single-herb traditional Chinese medicine (TCM) preparation derived from AM, approved for treating chronic kidney disease (CKD) and rheumatoid arthritis by the *National Medical Products Administration* of China (Luan et al., 2020; Wei et al., 2023). HKC is also commonly prescribed for conditions including primary chronic glomerulonephritis, nephrotic syndrome (NS), diabetic nephropathy (DN), hepatitis B-associated glomerulonephritis, and chronic renal failure (Luan et al., 2020; Wei et al., 2023). Similarly, Huangkui Lianchang decoction (HLD), formulated with AM as the principal ingredient, has shown efficacy in alleviating ulcerative colitis (UC) (Xu et al., 2024). Other AM-containing prescriptions—such as Jiahua Tablets, Chuangling Liquid, Huangkui Siwu Formula, Yu Kui Qing, Qikui Granules, Huangshu Kuihua paste, and Er Huang Ointment—have demonstrated therapeutic effects on CKD, skin ulcers, and chronic inflammatory diseases (Guo and Wang, 2017; Yan et al., 2018; Lu T. et al., 2020). This review synthesizes current reports on the ethnobotany, phytochemistry and their bioactive properties, pharmacological activities, and potential mechanisms of AM and its formulations. It also highlights limitations in existing research and discusses prospects for future applications, underscoring AM’s potential to bridge traditional and modern medicine.

## 2 Literature search methods

**FIGURE 1:**
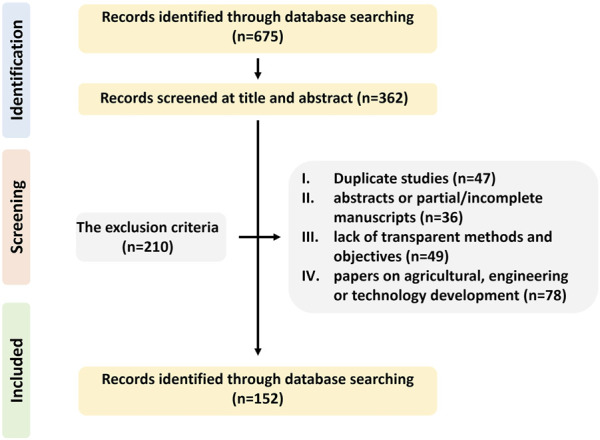
Flowchart of study selection for this review.

This review used the keywords “*A. manihot* (L.) Medicus” to search PubMed, Science Direct, Web of Science, Sarcandra, Baidu Scholar, Google Scholar, Connected Papers, Springer Search, and CNKI databases from 1981 to 2024, and 675 pieces of information were collected. This article aligns with the Preferred Reporting Items for Systematic Reviews and Meta-Analyses (PRISMA) guidelines (Page et al., 2021). The botanical information was obtained through the Flora of China (www.iplant.cn). The inclusion criteria for this review were the availability of relevant studies on botany, traditional uses, pharmacology, and TCM formulas of AM. This article included all published studies in Chinese and English, *in vitro* and *in vivo* trials, and clinical studies. Duplicate studies, abstracts or partial/incomplete manuscripts, lack of transparent methods and objectives, and papers on agricultural practices, engineering, and technology development were excluded (Figure 1).

## 3 Plant ethnobotany and distribution

**FIGURE 2:**
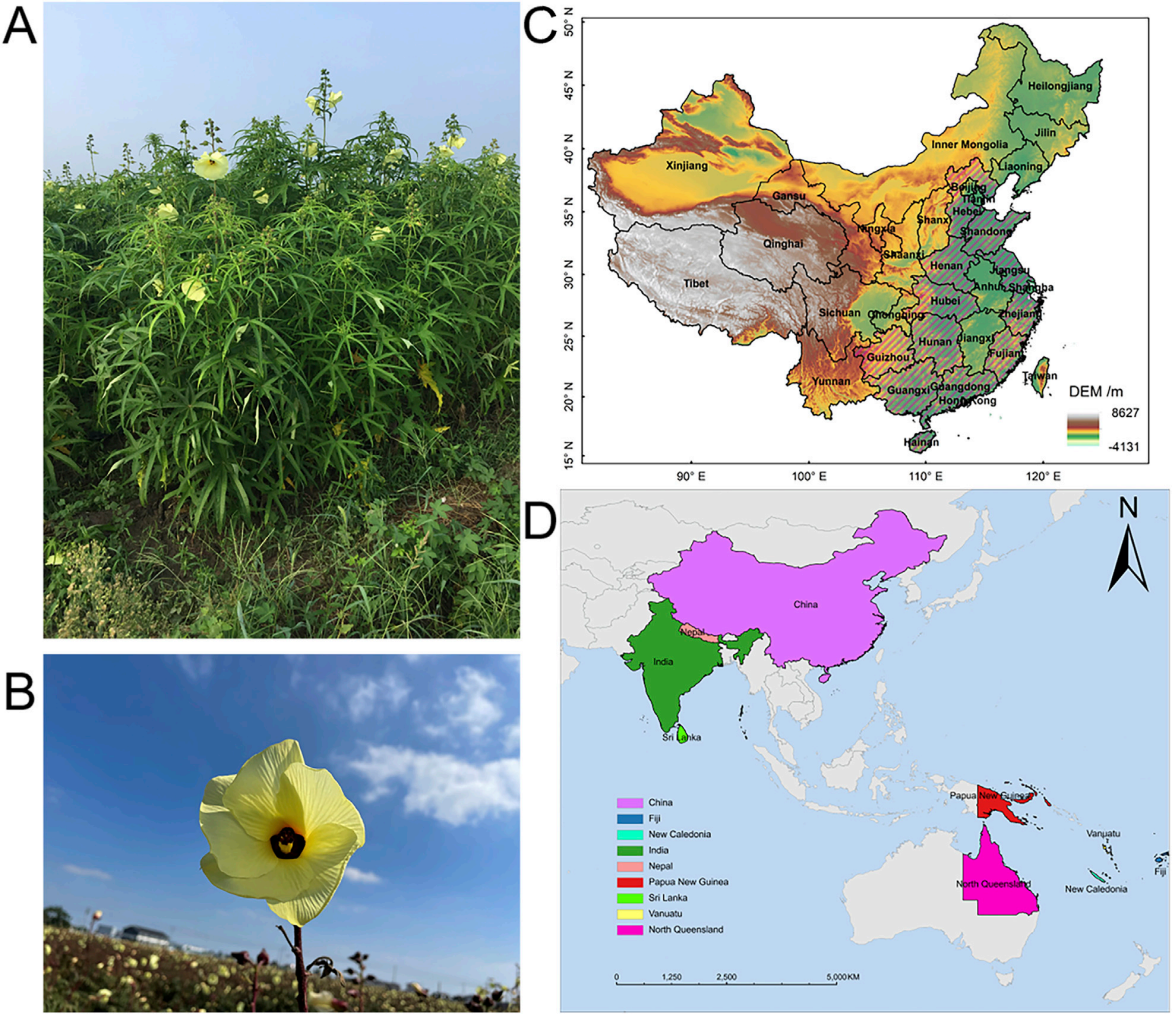
Plant morphology and natural distribution of *Abelmoschus manihot* (L.) Medicus. **(A,B)** Photographs of *Abelmoschus manihot* (L.). Medicus. **(C)** AM is native to provinces in China including Hebei, Shandong, Henan, Hubei, Hunan, Hainan, Guizhou, Guangxi, Guangdong, Jiangsu, Zhejiang, and Fujian, which are marked with slashes in figure. **(D)** AM is distributed in China, India, Nepal, Papua New Guinea, Vanuatu, Fiji, New Caledonia, Sri Lanka, and North Queensland.

*Abelmoschus manihot* (L.) Medicus (AM) is an annual or perennial robust, erect herb with vigorous growth of aboveground parts and a plant height of 1–2 m (Figure 2A). The root of AM is slightly conical, with many lateral roots (Shao, 2012). AM leaves are palmately divided and have irregular, coarsely toothed margins. The flower is divided into five petals; the periphery is yellowish or pale yellow, and the center is purplish. The flower diameter is 10–20 cm, flowering from bottom to top, and the ovary is 5-chambered (Figure 2B). The plant flowers from August to October and is considered highly ornamental value (Chen et al., 2016). The capsule is oblong, pointed, and hirsute, 5.0–7.5 cm long, and contains about 50 seeds per capsule. At maturity, the seeds are gray-black and kidney-shaped, with many stripes on the surface.

AM originated in the southern part of China. It prefers to be grown in warm and humid weather with abundant rainfall and adequate sunshine in good drainage and loose, fertile soil. However, it is adaptable and now is widely distributed in tropical and subtropical regions. The natural distribution of AM in China is mainly concentrated in plain areas, such as Hebei, Henan, Shandong, and Fujian (Figure 2C). AM is also distributed in India, Sri Lanka, North Queensland, and other countries (Figure 2D).

## 4 Phytochemistry and their bioactive properties

Chemical investigations have revealed that different parts of *A. manihot* (L.) Medicus (AM) contain distinct chemical components. The flowers are primarily rich in flavonoids, while the seeds are abundant in amino acids and unsaturated fatty acids. The roots, stems, and leaves mainly contain polysaccharides (Li Z. et al., 2016). While the chemical composition of AM has been well-studied, research into the pharmacological effects of its main components is still in the early stages.

### 4.1 Flavonoids

Flavonoids represent the primary chemical constituents and the most significant bioactive components of AM. Forty-eight flavonoids have been isolated and identified from AM flowers. These compounds can be broadly categorized into four groups: 1) Quercetin and its glycoside derivatives, including quercetin, rutin, hyperoside, quercetin-3′-O-β-D-glucoside, quercetin-3-O-β-D-glucoside, quercetin-3-O-β-D-6′-acetylglucopyranoside, quercetin-3-O-β-robinobioside, quercetin-3-O-β-rutinoside, and quercetin-3-O-β-D-xylopyranosyl (1→2)-β-D-galactopyranoside; 2) Gossypetin and its glycoside derivatives, such as gossypetin, hibifolin, and gossypetin-3′-O-β-D-glucoside; 3) Myricetin and its glycoside derivatives, exemplified by myricetin, myricetin-3-O-β-D-glucoside, cannabiscitrin, myricetin-3-O-β-D-galactopyranoside, myricetin-3-O-rutinoside, myricetin-3-O-robinobioside, and myricetin-3-O-β-D-xylopyranosyl-(1→2)-β-D-glucopyranoside; 4) Other flavonoid compounds, such as tiliroside, hibiscetin-3-O-glucoside, floramanoside B, floramanoside C, and 5-hydroxy-4′,7,8-trimethoxyflavone. Based on current literature, Table 1 summarizes the pharmacological effects and associated mechanisms of action for key representative flavonoid constituents of AM, including: hyperoside, quercetin, isoquercitrin, gossypetin-3′-O-β-D-glucoside, quercetin-3′-O-β-D-glucoside, gossypetin-8-O-β-D-glucuronide, myricetin-3-O-β-D-galactopyranoside, and abelmanihotols A-C.

**TABLE 1:** The pharmacological effects of the active ingredients of AM.

Compounds	Compounds type and composition	Content in AM	Pharmacological effect	Mechanism of action	Ref
Hyperoside	Flavonoids	Hyperoside is ≥0.5% in AM flowers and is required to be ≥1% in HKC	Anti-adipogenic effect Anti-inflammatory effect Anti-fibrotic effect Antioxidant effect Antidepressant effect Neuroprotective effect	Regulating NMDA receptors in the periaqueductal gray Inhibiting NLRP3 inflammasome Improving the function of vascular endothelium Myocardial SOD↑, oxygen free radicals↓, and improve the lipid metabolism disorder in diabetic mice Regulating the AMPK-ULK1 signaling pathway	Wang et al. (2022a), Fernando et al. (2024), Gao et al. (2024), Li et al. (2024a), Ogunro and Olasehinde (2024), Wang et al. (2024c), Xia et al. (2024), Gao et al. (2025), Zhao et al. (2025), Zhou et al. (2025)
Quercetin	Flavonoids	72.0 mg in 1000 mg total favone of AM flowers	Anti-inflammatory effect Antioxidant effect Antiplatelet effect Diuresis Glycolipid metabolism improvement	ROS↓, DDAH II↑ The level of ADMA↓, the content of NO and the ratio of p-eNOS/eNOS↑ Inhibiting the expression of ICAM-1, VCAM-1 and E-selectin to reduce the adhesion between endothelial cells and neutrophils Increase proliferation of EPCs through PI3K/AKT signaling pathway Inhibition of HIF-1a on the NEAT1/HMGB1 signaling pathway Inhibition non-enzymatic glycation and oxidative damage in the kidney of diabetic rats	Sun et al. (2019), Chen et al. (2020), Riemschneider et al. (2021), Luo et al. (2022), Zhang et al. (2022a), Topcu-Tarladacalisir et al. (2024), Zhao et al. (2024b), Abdou et al. (2025)
Isoquercitrin	Flavonoids	121.2 mg in 1000 mg total favone of AM flowers 0.0116–0.5064 mg in 1000 mg AM stems and leaves	Antioxidant effect Anti-inflammatory effect Anti-diabetic effect Anti-tumor effect Anti-apoptotic effect Neuroprotective properties	Inhibition oxidative stress and neuronal apoptosis via Nrf2-mediated NOX4/ROS/NF-κB signaling pathway Regulation the expression of CREB, Bax, Bcl-2, and caspase-3 to alleviate hippocampus neuron apoptosis Activating AMPK pathway and suppressing TGF-β signaling to alleviate hepatic lipid accumulation and oxidative stress suppressing the activation of TLR4, NF-κB and MAPK Mediation of the stimulatory effect on glucose uptake independent of insulin receptor activation through PI3K, MAPK, MEK/ERK pathways and *de novo* protein synthesis to GLUT-4 translocation Regulating the proliferation and differentiation of preadipocytes Regulation angiogenesis-relevant proteins, such as vasohibin-1 and vasohibin-2 expressions	Cai et al. (2016), Cao et al. (2017), Wang et al. (2017), Dai et al. (2018), Qin et al. (2018), Jayachandran et al. (2019), Jayachandran et al. (2020), Rey et al. (2020), Silva et al. (2022)
Gossypenin -3′-O-β-D-glucoside	Flavonoids	Not detected	Anti-steatotic and anti-fibrotic effects	Glutamic-pyruvic transaminase↓ Glutamic-oxaloacetic transaminase↓ SOD↑	Cheng et al. (2010), Chen et al. (2012a)
Quercetin-3′-glucoside	Flavonoids	≈4.5 mg/g in AM flowers and seeds (Yin et al., 2021)	Glycolipid metabolism improvement Anti-depressant activity	Glucose and lipid metabolism-related factors (PPARγ, C/EBPα, SREBP-1, adiponectin, lactone and resistin) ↑ The utilization of glucose↑ Improve insulin resistance The expression of BDNF↑	Cai et al. (2016), Cai et al. (2017b)
Gossypolin-8-O-β-glucuronic acid	Flavonoids	126.8 mg in 1000 mg total favone of AM flowers (Xue et al., 2023)	Glycolipid metabolism improvement Renal tubulointerstitial fibrosis improvement	Glucose and lipid metabolism-related factors (PPARγ, C/EBPα, SREBP-1, adiponectin, lactone and resistin) ↑ The utilization of glucose↑ Improve insulin resistance Inhibiting NADPH/ROS/ERK signaling pathway	Cai et al. (2016), Cai et al. (2017a)
Gossyptin-8-O-β-D-glucuronide	Flavonoids	Not detected	Anti-depressant activity	The expression of BDNF↑	Cai et al. (2017b)
Myricetin-3-O-β-D-galactopyranoside	Flavonoids	≈27 μg/g in AM roots, seeds and leaves ≈1.2 mg/g in AM flowers (Yin et al., 2021)	Antiphotoaging properties Anti-osteoporotic effect	Repressing MAPK/AP-1 signaling and stimulating the TGFβ/Smad signaling	Oh et al. (2020), Karadeniz et al. (2021)
Abelmanihotols A	Flavonoids	Not detected	Anti-inflammatory effect	Inhibiting NLRP3 inflammasome	Su et al. (2022)
Abelmanihotols B	Flavonoids	Not detected	Anti-inflammatory effect	Inhibiting NLRP3 inflammasome	Su et al. (2022)
Abelmanihotols C	Flavonoids	Not detected	Anti-inflammatory effect	Inhibiting NLRP3 inflammasome	Su et al. (2022)
AMPS-a	Polysaccharides (glucose, mannose, galactose, and fucose)	Not detected	Anti-tumor effect	Proliferation of hepatoma cells (SMMC-7721, HepG2) and gastric cancer cells (MGC-803, MKN-45) ↓	Zheng et al. (2016)
KSK-JT	Polysaccharides (arabinose, galactose, glucose, galacturonic acid, rhamnose, and mannose)	Not detected	Immunomodulatory activity	Proliferation of immune cells activates the phagocytosis of macrophages↑ The release of NO↑	Liu et al. (2016), Zheng et al. (2016)
S-SLAMP-a3	Polysaccharides (Mannose, rhamnose, glucuronic acid, glucose, galactose, and arabinose)	Not detected	Immunomodulator activity	Lymphocyte proliferation↑ The content of TNF-α and IL-6 in RAW264.7↑	Pan et al. (2018)
SLAMP-a	Polysaccharides (Mannose, rhamnose, glucuronic acid, glucose, galactose, and arabinose)	Not detected	Immunomodulatory activity	Lymphocyte proliferation↑ The content of TNF-α and IL-6 in RAW264.7↑	Pan et al. (2018)
SLAMP-c	Polysaccharides (Mannose, rhamnose, glucuronic acid, glucose, galactose, and arabinose)	Not detected	Immunomodulatory activity	Lymphocyte proliferation↑ The content of TNF-α and IL-6 in RAW264.7↑	Pan et al. (2018)
SLAMP-d	Polysaccharides (Mannose, rhamnose, glucuronic acid, glucose, galactose, and arabinose)	Not detected	Immunomodulatory Activity	Lymphocyte proliferation↑ The content of TNF-α and IL-6 in RAW264.7↑	Pan et al. (2018)

### 4.2 Polysaccharides

AM flowers, roots, stems, and leaves are rich in soluble polysaccharides. Notably, the total polysaccharide content in the stems can reach as high as 10.86% (Li et al., 2025). Recent studies have confirmed that AM polysaccharides possess skincare, immunomodulatory, and anti-tumor activities (Liu et al., 2016; Pan et al., 2018; Wang J. et al., 2024). Specific polysaccharide fractions—such as AMPS-a, KSK-JT, S-SLAMP-a3, SLAMP-c, and SLAMP-d—contribute significantly to these immunomodulatory and anti-tumor activities (Table 1).

### 4.3 Other compounds

A total of 22 amino acids and 16 nucleosides have been identified in AM. Among these, the amino acid content is higher in the flowers, reaching 4.737 mg/g, while nucleoside levels in the leaves are relatively lower at 1.474 mg/g (Liu et al., 2016). Leaf composition analysis reveals 1.77% lipids, 2.20% protein, 1.61% ash, and 88.4% moisture. Steroids and triterpenoids have also been detected in AM stems (Wen et al., 2015).

AM seeds are rich in fatty acids, soluble polysaccharides, soluble proteins, amino acids, nucleosides, and mineral elements (Liu et al., 2012; Wen et al., 2015). Total fatty acids constitute 10.22% of seed composition, with unsaturated fatty acids accounting for 78.01%–79.40% of this fraction (Liu et al., 2016). Amino acids are abundant (10.08%–10.15%), and essential amino acids represent 38.42%–39.40% of total free amino acids (Liu et al., 2016). Nucleoside content is relatively low (3.01–3.11 mg/g) (Liu et al., 2016). AM seeds also contain a diverse profile of 24 mineral elements—including K, Ca, Fe, Mn, Cu, Zn, and Mo—with levels of harmful elements (Hg, As, Cd) below food hygiene standards (Wen et al., 2015). Studies report that unsaturated fatty acids from AM seeds reduce serum levels of total cholesterol (TC), triglycerides (TG), alanine aminotransferase (ALT), and aspartate aminotransferase (AST) in hyperlipidemic rats, while increasing high-density lipoprotein (HDL) (Wu, 2011). These findings demonstrate the significant nutritional and medicinal value of AM seeds.

## 5 Pharmacological activities of AM

**FIGURE 3:**
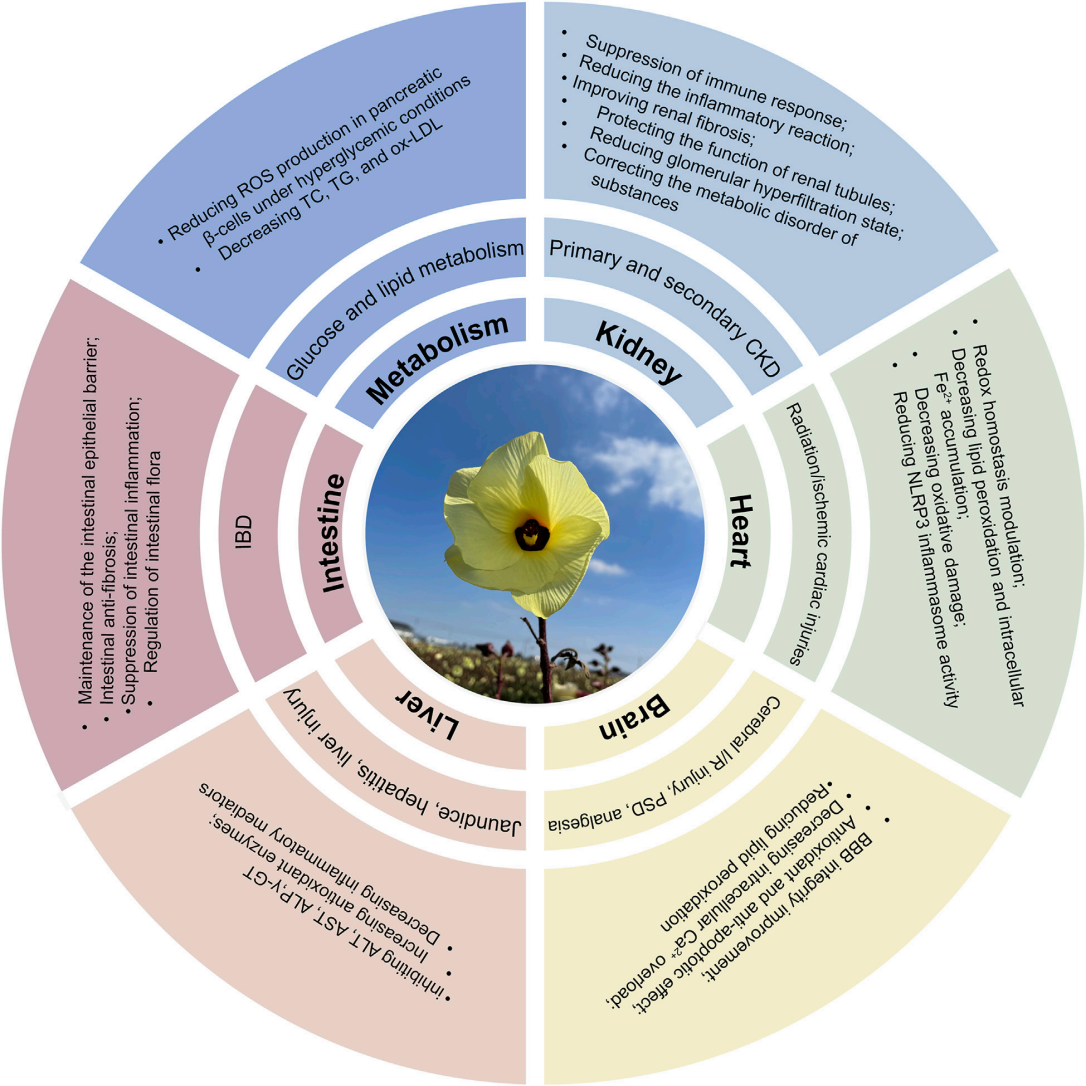
Multifunctional mechanisms of AM in protecting multiple organs and treating diseases.

**FIGURE 4:**
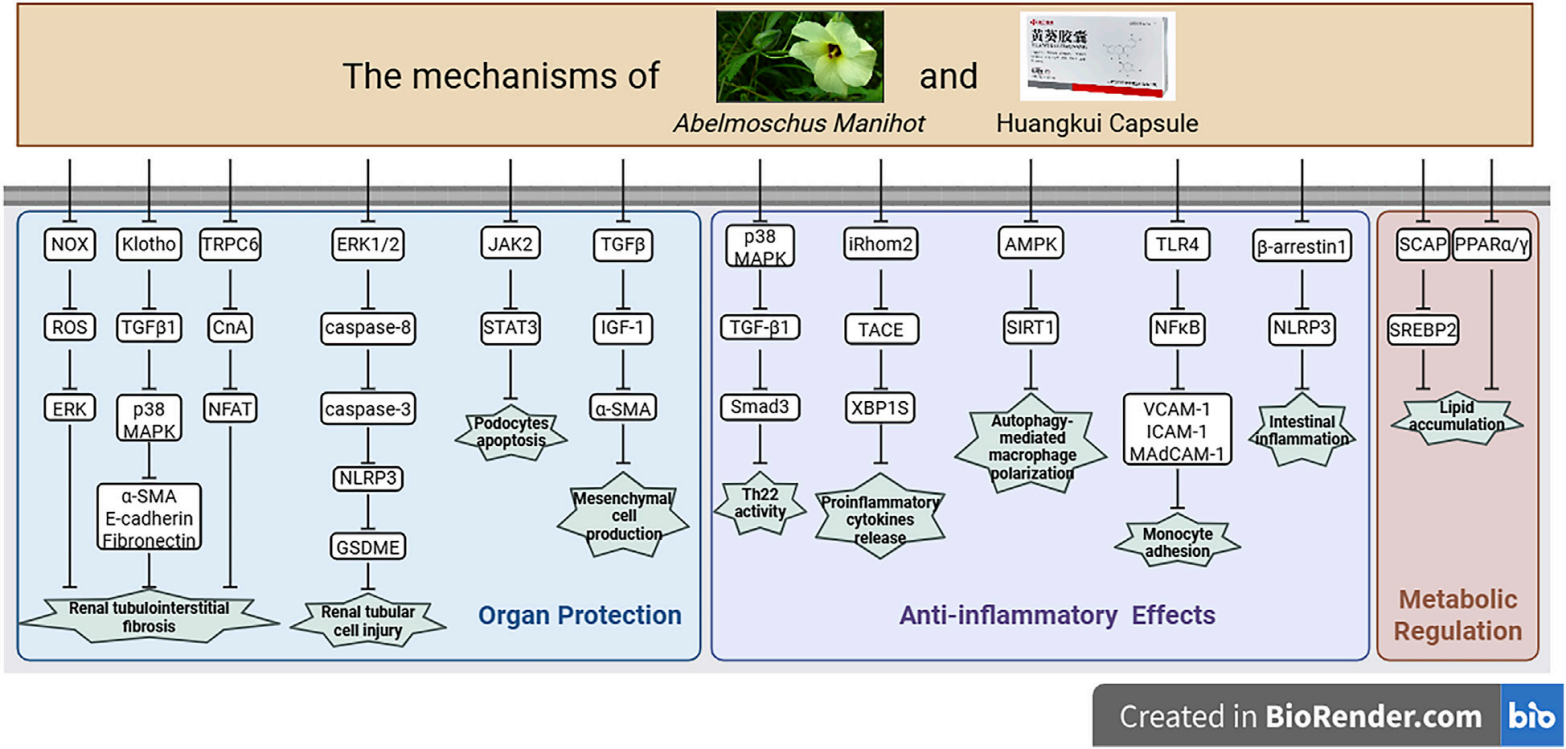
Potential mechanisms of AM or HKC in disease treatment, created with BioRender.com. This figure summarizes the multi-target mechanisms of AM in organ protection, anti-inflammatory effects, and metabolic regulation. The mechanisms include: organ protection via inhibiting renal tubulointerstitial fibrosis by regulating the NOX/ROS/ERK pathway (e.g., α-SMA, E-cadherin), reducing podocyte apoptosis through JAK2/STAT3, suppressing mesangial cell generation via TGFβ/IGF-1, and alleviating renal tubular cell injury through the ERK1/2/caspase pathway and NLRP3/GSDME; anti-inflammatory effects by suppressing inflammatory activity through p38 MAPK/TGFβ/Smad3, decreasing pro-inflammatory cytokine release via iRhom2/TACE/XBP1S, regulating macrophage polarization via AMPK/SIRT1, and inhibiting monocyte adhesion through TLR4/NFκB and β-arrestin1/NLRP3; as well as metabolic regulation through ameliorating lipid accumulation via the SCAP/SREBP2 and PPARα/γ pathways.

*Abelmoschus manihot* (L.) Medicus (AM), particularly its flowers, exhibits therapeutic potential against various diseases including CKD, IBD, ischemic cardiac/cerebral injuries, hepatic injury, and diabetes mellitus (Figure 3). Advances in research methodologies have enabled systematic investigation into the pharmacological activities of AM. This section summarizes the established pharmacology and underlying mechanisms of AM and HKC (Table 2; Figure 4).

**TABLE 2:** Summary of the pharmacological activities of AM.

Biological activity	Study type	Extract/Compound	Testing subject	Mechanisms/Effects	Dosage	Ref
Anti-inflammatory effects	*In vivo*	Flowers; 70% ethanol extract	LPS-induced cystitis in mice	The expression of TLR4, MYD88, IκBα, p-IκBα, NF-κB p65, and p-NF-κB p65↓	0.75, 1.5, 3 g/kg, *i.g*	Zhou et al. (2022)
Anti-inflammatory effects	*In vivo*	AM Seeds	Collagen-induced rat arthritis	The expression of IFN-γ, IL-6, IL-17, IL-1β, and TNF-α↓; the expression of IL-10, IL-4 ↑ the protein Bcl-2/Bax, STAT3, and JAK2 levels↓ the expression of Caspase3, SOCS1, and SOCS3 in the JAK2/STAT3 pathway ↑	157.5, 315, 630 mg/kg, *i.g*	Tao et al. (2024b)
Anti-inflammatory effects	*In vitro*	A mixed neutral polysaccharide (SLAMP-a) and two acidic polysaccharides (SLAMP-c and SLAMP-d) were obtained from stems and leaves of AM.	Spleen lymphocyte proliferation assay *in vitro* Cytotoxicity assay and nitric oxide assay on RAW264.7	S-SLAMP-a3, SLAMP-c and SLAMP-d exhibited significant immunomodulatory activity, while SLAMP-a showed little effects.	50, 100, and 200 μg/mL *in vitro*	Pan et al. (2018)
Anti-inflammatory effects	*In vitro*	Abelmanihotols A−C (1–3) from AM seeds	LPS-induced NO release in THP-1 cells	AM blocked the formation of NLRP3 inflammasome formation bysuppressing apoptosis-associated speck-like protein oligomerization, thereby attenuating caspase-1 activation and IL-1β release	10 μΜ*in vitro*	Su et al. (2022)
Anti-inflammatory effects	*In vivo,* *in vitro*	Total flavones of AM flowers	Influenza A virus-induced lung inflammation	Inflammatory responses↓ MAPK signaling pathway↓; viral eradiation↑	125, 250, 500 mg/kg, *i.g* 25, 50, 100 μg/mL *in vitro*	Gao et al. (2022)
Anti-inflammatory and anti-oxidative effects	*In vivo,* *in vitro*	HKC	LPS-Induced Acute Lung Injury and Macrophage Activation	Glutathione peroxidase and catalase activities↑; the expression of miR-451↑ the production of nitric oxide, TNF-α, and IL-6↓	150, 300, and 600 mg/kg, *i.g* 25, 50, 100, 200 μg/mL	Deng et al. (2021)
Anti-inflammatory effects	*In vivo*	Flowers; 80% ethanol extract	DSS-induced UC	UC signs, symptoms, colon macroscopic lesion scores, and disease activity index (DAI) scores↓; the levels of proinflammatory cytokines (IL-6, IL-1β, IL-18, IL-17, and TNF-α) ↓ The mRNA expression levels of NLRP3, ASC, and caspase 1 in colon tissue ↓; the expression of occludin-1, claudin-1, and ZO-1↑	2.05, 4.1, 8.2 g/kg, *i.g*	Wu et al. (2021)
Anti-inflammatory effects	*In vivo,* *in vitro*	Total flavones of AM flowers	TNBS-induced Colitis in mice LPS-induced RAW264.7 cells	The levels of cytokines in the serum↓ MPO activity in the colon tissues↓ NF-κB and MAPK signaling pathways↓	125, 250 and 500 mg/kg, *i.g* 50, 100, 200 μg/mL *in vitro*	Zhang et al. (2019a)
Anti-inflammatory effects	*In vivo,* *in vitro*	Total flavones of AM flowers	the chronic renal failure rat models were induced by uninephrectomy, potassium oxonate, and proinflammatory diet LPS-induced RAW 264.7 cells	Renal dysfunction and renal tubulointerstitial lesions↓; the content of *Bacteroidales* and *Lactobacillales*↓ the content of *Erysipelotrichales*↑ modulation of macrophage polarization, including markers of M1/M2 macrophages TFA reversed the expression of BECN1 and phosphorylation of p62 protein and LC3 conversion by activating the AMPK-SIRT1 signaling	136 mg/kg, *i.g* 20 μg/mL *in vitro*	Tu et al. (2020)
Anti-inflammatory effects	*In vivo,* *in vitro*	Total flavones of AM flowers	DSS-induced colitis TNF-α-induced MAECs	DAI score, colon shortening, and histological injuries↓; the expression of cytokines (IL-1β and TNF-α) and adhesion molecules (ICAM-1, VCAM-1, and MAdCAM-1) ↓ the phosphorylation and nuclear translocation of NF-κB in MAECs↓	30, 60, 120 mg/kg, *i.g* 10, 50 μg/mL *in vitro*	Xue et al. (2023)
Anti-inflammatory effects and Regulation of Intestinal Flora	*In vivo*	Flowers; 75% ethanol extract	DSS-induced colitis in mice	Microbial diversity↑; the abundance of short chain fatty acids (SCFAs)-producing gut microbiota↑ Treg generation↑ and Th17 development↓	0.25, 0.5, 1 mg/g, *i.g*	Zhang et al. (2019b)
Regulation of Intestinal Flora	*In vivo*	HKC	non-obese diabetes mice with DN	*Faecalitalea* and *Muribaculum* ↑ *Phyllobacterium*, *Weissella* and *Akkermansia* ↓	0.45 g/kg, *i.g*	Shi et al. (2023)
Regulation of Intestinal Flora	*In vivo*	Total flavones of AM flowers	DSS-induced colitis	*Akkermansia muciniphila* (*A. muciniphila*)↑; colonic inflammatory response and intestinal epithelial barrier dysfunction↓	62.5, 125 mg/kg, *i.g*	Bu et al. (2021)
Regulation of Intestinal Flora	*In vivo*	Total flavones of AM flowers	DSS-induced colitis; chronic stress-induced depression	TFA treatment improved the depression-like phenotype, the disturbed gut microbiota, and the intestinal barrier function in chronic stress mice	62.5, 125 mg/kg, *i.g*	Wang et al. (2021)
Renal protective effect	*In vivo*	Total flavones of AM flowers	db/db mice	Urinary albumin-to-creatinine ratio ↓ The expression of *slc2a2*, *slc4a1*, *slc5a2*, *slc5a3*, *slc5a8*, *slc6a20*, *slc27a2*, *slc12a3*, *slc34a1* and *slc38a2*↑	0.076 g/kg, *i.g*	Yu et al. (2023a)
Renal protective effect	*In vivo*	HKC	db/db mice	The urinary albumin-to-creatinine ratio↓ The activities of col4a3, slc5a2, slc34a1, slc12a3, and slc4a1↓	0.84 g/kg, *i.g*	Yu et al. (2023b)
Renal protective effect	*In vivo*	HKC	Immunoglobulin A nephropathy rat model	TGF-b1/Smad3 signaling pathway↓ CCL20, CCL22, and CCL27 levels↓	2, 5 g/kg, *i.g*	Pei and Li (2021)
Renal protective effect	*In vivo*	Total flavones of AM flowers	db/db mice	In db/db mice administered with HKC and TFA, 7 flavonoid prototypes and 38 metabolites were identified	HKC (0.84 g/kg) and TFA (0.076 g/kg), *i.g*	Diao et al. (2024)
Renal protective effect	*In vitro*	Total flavones of AM flowers	Iopromide induced renal tubular cell injury	Iopromide induced renal tubular cell injury and apoptosis ↓ The phosphorylation of AKT↑	0.6 mg/mL *in vitro*	Xu et al. (2022)
Renal protective effect	*In vivo*	Total flavones of AM flowers	Streptozotocin-induced DN	The urinary microalbumin to creatinine ratio and 24-h urinary total protein↓; glomerular cell apoptosis↓	200 mg/kg, *i.g*	Zhou et al. (2012)
Renal protective effect	*In vivo*	Flower or leaf extracts of AM	A DN model by combining unilateral nephrectomy, a high-fat diet, and streptozotocin in C57BL/6 mice	Hepatic injury, proinflammatory cytokines, and lipid accumulation↓; the expression of proteins by regulating autophagy and mitochondrial dynamics↑	100 mg/kg, *i.g*	Kim et al. (2018)
Renal protective effect	*In vivo,* *in vitro*	Total flavones of AM flowers	a DKD rat model and the NRK-52E cells	TFA improved biochemical parameters, renal tubular injury, and ferroptosis in the DKD rats. TFA inhibited ferroptosis by ameliorating iron deposition, lipid peroxidation capacity, and ferroptosis-related proteins expression *in vitro*	136 mg/kg TFA suspension, *i.g* 50, 100, 150, 200, 250 μg/mL *in vitro*	Wang et al. (2023b)
Renal protective effect	*In vivo,* *in vitro*	Flowers; 75% ethanol extract	Adriamycin-induced NRK-52E cells Adriamycin-induced nephropathy in rats	TEA ameliorated Adriamycin-induced cellular morphological changes, cell viability, and apoptosis TEA suppressed NLRP3 inflammasomes via inhibition of ERK1/2 signal transduction	1.5 g/kg, *i.g* 100 μg/mL *in vitro*	Li et al. (2019)
Renal protective effect	*In vivo,* *in vitro*	Total flavones of AM flowers	DN rats via unilateral nephrectomy and intraperitoneal injection of streptozotocin AGEs-induced HK-2 injury	IC_50_ of TFA is 35.6 µM in HK2 and 39.6 µM in HRMC; the activation of iRhom2/TACE signalling↓ the expression of proinflammatory cytokines↓	300, 135 and 75 mg/kg, *i.g* 20 μg/mL *in vitro*	Liu et al. (2017)
Renal protective effect	*In vitro*	Total flavones of AM flowers	MPC-5 cells under high glucose (HG) conditions	The protein expression levels of gasdermin D, interleukin-1β, and interleukin-18↓; the protein expression levels of nephrin, ZO-1, WT1 and podocalyxin↑ the protein levels of NIMA-related kinase7, NLRP3, ASC, and caspase-1↓ the protein expression levels of p-PI3K and p-Akt↑	5, 10, and 20 μg/mL *in vitro*	Liu et al. (2021a)
Gastroprotective Activity	*In vivo*	Total flavones of AM flowers	Ethanol-induced gastric ulcer	The activity of SOD and GSH↑; the levels of MDA↓ the levels of Bax, TNF-α, and NF-*κ*B (p65) expressions↓ the Bcl-2 expression level↑	300, 600, and 1200 mg/kg, *i.g*	Zhang et al. (2020)
Hepatoprotective effect	*In vivo,* *in vitro*	Total flavones of AM flowers	Carbon tetrachloride (CCl_4_) induced hepatocyte damage *in vitro* and liver injury *in vivo*	Levels of ALT, AST and ALP↓; the MDA level ↓ and the content of GSH ↑ in the liver activities of antioxidative enzymes (SOD, GPx, CAT and GST) ↑ the inflammatory mediators (TNF-α, IL-1β and NO) ↓	125, 250 and 500 mg/kg, *i.g* 4.5–72 mg/L *in vitro*	Ai et al. (2013)
Hepatoprotective effect	*In vivo*	Total flavones of AM flowers	α-naphthylisothiocyanate-induced cholestatic liver injury in rats	Levels of ALT, AST, LDH, ALP, GGT, TBIL, DBIL and TBA↓; polymorphonuclear neutrophil in-filtration and histological damages↓	125, 250 and 500 mg/kg, *i.g*	Yan et al. (2015)
Cardioprotective effect	*In vivo*	Total flavones of AM flowers	myocardial ischemia/reperfusion in rats	Myocardial infarction area, serum creatinine kinase, LDH levels, serum IL-6, IL-1β and TNF-α production↓; the activities of SOD↑ and the amounts of MDA↓ NLRPR3 inflammasome↓	40, 80 mg/kg, *i.g*	Lv et al. (2017)
Neuroprotective actions	*In vitro*	Total flavones of AM flowers	cultured rat hippocampal neurons	TFA rapidly and reversibly inhibited the I_NMDA_ in a concentration-dependent manner TFA non-competitively inhibited the INMDA by enhancement of the NMDA receptor desensitization Intracellular application of TFA did not alter the TFA inhibition of I_NMDA_.	0.2, 0.8 mg/mL *in vitro*	Cheng et al. (2006)
Neuroprotective Effect	*In vitro*	Flowers; 95% ethanol extract	H_2_O_2_-induced cytotoxicity, oxidative stress and inflammation in PC12 cells	The pro-inflammatory cytokines and mediators (TNF-α, IL-1β, IL-6, COX-2 and iNOS) ↓; the production of nucleotide excision repair (NER)-related proteins↑	125, 250, 500 μg/mL *in vitro*	Wang et al. (2022c)
Neuroprotective Effect	*In vivo*	Total flavones of AM flowers	Cerebral ischemic reperfusion injury in rats	Serum LDH activity and MDA level↓	20, 40, 80, 160 mg/kg, *i.g*	Wen and Chen (2006)
Neuroprotective Effect	*In vivo*	Total flavones of AM flowers	Poststroke Depression Injury in Mice	Escape-directed behavioral impairment induced by PSD MDA levels↓; the activity of SOD, GSH-Px↑ neuronal death/losses ↓ BDNF both at mRNA and protein levels↑ CREB mRNA levels↑	40, 80, 160 mg/kg, *i.g*	Liu et al. (2009)
Anticonvulsant, Atidepressant	*In vivo*	Flowers; 75% ethanol extract	PTZ-induced clonic convulsions and mortality	Immobility time in the FST in mice↓ Fiveparent components including isoquercitrin, hyperoside, hibifolin, quercetin-3-O-glucoside, quercetin and threemetabolites were detected in rat brain	50, 100, 200 mg/kg, *i.g*	Guo et al. (2011)
Anti-cancer	*In vivo*	Flowers; 75% ethanol extract	Multiple Myeloma in Mouse Model	Survival rate↑	3.75 g/kg, 3 times/week, *i.g*	Hou et al. (2020)
Metabolic Regulation	*In vivo*	Flowers; 75% ethanol extract	Adriamycin -induced CKD model rats	Deglycosylation and methylation are the major metabolic pathways	3 g/kg, *i.g*	Du et al. (2017)
Sex hormones Regulation	*In vivo*	Flowers; 75% ethanol extract	Wild-type adult zebrafish	The expression levels of sex-related genes (*lhcgr*, *ar*, *cyp19a1a*, and *cyp19a1b*) ↑ The chasing number, fertilized egg production, and hatching rate↑	0.2%, 1%, 10% extract diet	Chang et al. (2022)
Antioxidant and Anti-Adipogenic Activity	*In vitro*	Total flavones of AM flowers	DPPH free radical-scavenging assay 3T3-L1 cell line	IC_50_ = 0.288 mg/mL The expression of PPARγ and C/EBPα↓	25, 50, 100, and 200 μg/mL *in vitro*	Cai et al. (2016)
anti-oxidative effects	*In vivo,* *in vitro*	Ethyl acetate fraction of AM flowers	H_2_O_2_-induced HepG2 cells and D-galactose-induced aging mice	Viability of H_2_O_2_-induced HepG2 cells↑; the ROS level, apoptotic cells, and activities of caspase 3/9↓ the levels of SOD and GSH-Px ↑ MDA generation and LDH release↓	25, 50, 100 mg/kg, *i.g* 15.6–1000 μg/mL *in vitro*	Liu et al. (2021b)
anti-oxidative effects	*In vivo*	Total flavones of AM flowers	D-galactose-induced oxidative stress in mouse liver	Antioxidant enzymes (CAT, GPx, SOD, and T-AOC) ↑ MDA production↓; expression of Nrf2 and its target antioxidants (HO-1 and NQO1)↑	40, 80, and 160 mg/kg, *i.g*	Qiu et al. (2017)
Anti-cancer effect	*In vitro*	AMPS-a (from the ethanol-extracted debris of AM flowers)	Hepatic (SMMC-7721, HepG2) and gastric (MGC-803, MKN-45) cancer cells	AMPS-a exhibited potent inhibitory effects on the proliferation of hepatic and gastric cancer cells	25, 50, 100, 200, 400 μg/mL *in vitro*	Zheng et al. (2016)
Bone loss improvement	*In vivo*	leaves of AM	Osteopenia induced by ovariectomy in rats	Bone mineral density↑; bone mineral content↑	10% (2.2 g/day/rat; 10% leaves) or 15% leaves (3.3 g/day/rat; 15% leaves) of AM	Puel et al. (2005)
Anti-fibrosis effect	*In vivo*	Total flavones of AM flowers	(TNBS)-induced chronic colonic inflammation	Body weight loss, colon length shortening, the morphological damage index score, and inflammatory response↓; the colonic expression of col1a2, col3a2, and hydroxyproline↓ α-SMA, TGF-β, vimentin, TIMP-1 expression↓	250 mg/kg, *i.g*	Qiao et al. (2021)
Pro-angiogenic ability	*In vivo,* *in vitro*	Total flavones of AM flowers	HUVECs *in vitro*; chick chorioallantoic membrane (CAM) *in vivo*	TFA promoted HUVECs proliferation TFA increased HUVECs migratory ability and the number of tubular structure, promoted vessel formation in HUVECs culture and CAM model; the expression of VEGF and KDR↑	5, 10, 20 μg/mL *in vitro*	Tang et al. (2017)
Pro-angiogenic ability	*In vitro*	Total flavones of AM flowers	HUVEC	Cell viability, wounding healing, transwell invasion, tube formation↑ PI3K and Akt phosphorylation↑ VEGF-A and VEGFR2 ex pression↑	5, 10, and 20 μg/mL *in vitro*	Zhu et al. (2018)

### 5.1 Anti-inflammatory and immunomodulatory effects

Inflammation accelerates organ injury through multiple mechanisms, while targeted anti-inflammatory intervention can significantly delay the progression of the disease associated with the inflammation. Current evidence demonstrates that AM downregulates pivotal inflammatory mediators—including M1 macrophage-derived cytokines (IL-1β, IL-12, IL-6, MMP-12), chemokines (MCP-1), and adhesion molecules (VCAM-1)—in diabetic nephropathy (DN) models (Ge et al., 2016; Gu et al., 2020). This anti-inflammatory action appears central to AM’s therapeutic mechanism, with additional research revealing its ability to modulate ER stress through iRhom2/tumor necrosis factor-α converting enzyme (TACE)/Spliced X-box binding protein-1 (XBP1S) pathways and regulate autophagy via adenosine monophosphate-activated protein kinase (AMPK)- sirtuin 1 (SIRT1) signaling (Liu et al., 2017; Tu et al., 2020). Parallel mechanistic studies reveal that HKC attenuates inflammatory cell activation and infiltration via p38 mitogen-activated protein kinase (MAPK) pathway inhibition, thereby reducing transforming growth factor beta 1 (TGF-β1) expression in nephropathy models (Chen P. et al., 2012). Current evidence establishes AM and HKC as renal protective agents targeting pathogenic T-lymphocyte subsets including Th22 cells, with single-cell RNA sequencing revealing critical renal receptor networks mediating HKC’s suppression of T-cell activation and infiltration (Wu et al., 2024). Simultaneously, these interventions modulate TGF-β1/Smad family member 3 (Smad3) signaling to reduce immune complex deposition in IgA nephropathy (IgAN) and mesangial proliferative glomerulonephritis (MsPGN) (Pei and Li, 2021; Wu et al., 2024), while enhancing erythrocyte-mediated clearance via redistribution of circulating complexes to erythrocyte surfaces—a mechanism shown to limit renal deposition (Hu et al., 2011). These multimodal actions collectively substantiate HKC’s clinical efficacy in IgAN through integrated immunoregulation (Pei and Li, 2021).

In addition, intestinal inflammation drives IBD progression by mediating tissue damage, fibrosis, and carcinogenesis. In 2,4,6-trinitrobenzenesulfonic acid solution (TNBS)/dextran sodium sulfate (DSS)-induced colitis models, AM ethanol extract attenuates pro-inflammatory cytokines (IL-6, IL-1β, IL-18, IL-17, TNF-α) and reduced myeloperoxidase activity in serum and colonic tissues (Cheng et al., 2015; Xue et al., 2023). Mechanistically, AM suppresses Nod-like receptor protein 3 (NLRP3) inflammasome activation via β-arrestin1 inhibition—evidenced by reducing NLRP3/apoptosis-associated speck-like protein containing caspases recruitment domain (ASC)/caspase-1 expression (Wu et al., 2021). Immune modulation studies revealed AM’s capacity to normalize T helper 17 cell (Th17)/regulatory T cell (Treg) balance through peroxisome proliferator-activated receptor gamma (PPAR-γ)-mediated enhancement of Treg cytokines (IL-10, TGF-β) with concurrent Th17 marker suppression (Qiao et al., 2021). *In vitro* studies confirmed AM’s inhibition of nuclear factor kappa B (NF-κB) and MAPK signaling in lipopolysaccharide (LPS)-stimulated macrophages (Zhang D. et al., 2019). Immune cell infiltration is critical in initiating and propagating the immune response against pathogens during the progression of UC (Neurath, 2014). This process is tightly regulated by adhesion molecules that mediate interactions between vascular endothelial cells and immune cells (Besendorf et al., 2022). Recent work identified the total flavonoids of AM (TFA) suppression of tumor necrosis factor-alpha (TNF-α)-induced NF-κB activation in colonic endothelial cells, thereby reducing vascular adhesion molecule expression (ICAM-1, VCAM-1, MAdCAM-1) and monocyte recruitment (Xue et al., 2023).

### 5.2 Anti-fibrotic effect

When tissues are damaged (such as due to infection, toxins, ischemia, mechanical injury, or chronic inflammation), the body activates its repair mechanism. The core of fibrosis lies in the activation and proliferation of fibroblasts. By differentiating into myofibroblasts with strong contractile and synthetic capabilities, they produce and secrete extracellular matrix components, thereby isolating the damaged area and preventing the spread of inflammation (Wang et al., 2025). However, as the injury stimulus persists or the repair process becomes imbalanced, the fibrotic response will proceed excessively, persistently and eventually lead to the aggravation of the disease state.

Renal fibrosis, representing the terminal pathological endpoint in CKD, is driven primarily by myofibroblast activation triggered by inflammatory mediators (TNF-α, IL-1β), metabolic disturbances (high glucose/lipid-induced reactive oxygen species (ROS)), mechanical stress, or hypoxia. These insults activate key signaling pathways (such as TGF-β/Smad3) to induce phenotypic transformation. In DN models, HKC suppresses TGF-β1 expression and reduces α-SMA levels (Gu et al., 2021). Furthermore, in unilateral ureteral obstruction models, HKC targets calcium-permeable TRPC channels, specifically modulating TRPC6/NFAT signaling. This mechanism is critical, as the protective effect of HKC is abolished in TRPC6-knockout mice (Gu et al., 2020). The protective role of HKC in renal disease is primarily mediated through the critical TRPC6/NFAT pathway, as complemented by its suppression of the TGF-β1/α-SMA axis. In addition, mechanistic studies reveal HKC’s capacity to inhibit fibrogenesis through coordinated p38 MAPK/phosphoinositide 3-kinase (PI3K)/protein kinase B (Akt) pathway downregulation (Mao et al., 2015), NLRP3 inflammasome suppression with toll-like receptor 4 (TLR4)/NF-κB blockade to prevent epithelial-mesenchymal transition (Han et al., 2019), NOX/ROS/ERK axis modulation (Li et al., 2019), and angiogenic receptor (VEGFR, PDGFR) inhibition coupled with pericyte myofibroblast transdifferentiation suppression (Wang M. et al., 2022). These actions are further complemented by connective tissue growth factor/osteopontin reduction via Smad7/SnoN upregulation and Klotho/TGF-β1/p38 pathway normalization in renal tubular cells (Gu et al., 2021). These studies collectively demonstrate that HKC orchestrates a coordinated suppression of critical pro-fibrotic pathways—including p38 MAPK/PI3K/Akt, TLR4/NF-κB/NLRP3, and NOX/ROS/ERK—thereby counteracting fibrogenesis at multiple levels.

Intestinal fibrosis—a pivotal complication in IBD progression with over 50% incidence in Crohn’s disease—drives intestinal stenosis, obstruction, and surgical intervention through excessive ECM deposition and pathological myofibroblast activation. AM counteracts this remodeling by reducing TNBS-induced weight loss, disease activity index (DAI) scores, and collagen deposition while improving histopathological features (Zhang et al., 2017). Mechanistically, AM suppresses fibrotic markers (col1a2, col3a2, α-SMA) via dual inhibition of TGF-β and IGF-1 signaling (Qiao et al., 2021; Zhang et al., 2024), remodels ECM through altered MMP/TIMP balance (elevated MMP-9/MMP-2 with suppressed TIMP-1), and attenuates TGF-β1-induced epithelial-mesenchymal transition (EMT) by blocking Smad2/3 phosphorylation and MAPK cascades (Yang et al., 2018). *In vitro* evidence confirms AMPK/mTOR-regulated autophagy mediates AM’s inhibition of IGF-1-driven collagen synthesis in intestinal fibroblasts (Zhang H. et al., 2022), establishing a multi-target therapeutic profile against fibrogenesis.

### 5.3 Metabolic and anti-oxidant regulation

The ethanol extract showed superior efficacy and reduced hyperglycemia-induced ROS in pancreatic β-cells (Wang S. W. et al., 2024). AM total flavonoids, TFA, enhanced glucose metabolism through dose-dependent promotion of glucose uptake in 3T3-L1 adipocytes and exerted hypoglycemic effects in diabetic murine models. TFA administration elevated superoxide dismutase (SOD) activity while reducing malondialdehyde (MDA) levels in diabetic rats, suggesting β-cell protection against oxidative damage (Zhou and Chen, 2016).

The ethanol extract of AM inhibited TG accumulation in 3T3-L1 adipocytes and lowered serum TC, TG, low-density lipoprotein cholesterol (LDL-C), and ox-LDL levels in hyperlipidemic rats, achieved through downregulation of PPARγ and CCAAT/enhancer-binding protein alpha (C/EBPα) mRNA to inhibit adipogenesis (An et al., 2011; Li J. et al., 2016). At the molecular level, AM ethanol extract orchestrates lipid homeostasis by normalizing adipogenic regulators (PPARα/γ, C/EBPα, SREBP-1), correcting adipokine imbalances (adiponectin/resistin), and alleviating ER stress-induced lipid dysmetabolism. Synergistically, TFA combined with glipizide reduced plasma lipids, blood urea nitrogen (BUN), creatinine, urinary microalbumin, and blood viscosity in DN rats (Li et al., 2018). Metabolic profiling reveals HKC’s systemic modulation of circulating metabolites including methionine sulfoxide, branched-chain amino acids, and cis-7-hexadecenoic acid (Shi et al., 2023). Complementary renal protection involves improved sodium handling via inhibition of renal medullary Na^+^-K^+^-ATPase activity, reducing pathological edema and lipid deposition in diabetic kidneys (Cai et al., 2016; Li et al., 2018; Qian et al., 2023).

AM also has a significant antioxidant effect. AM accelerates scald wound healing by modulating MDA/SOD via nuclear factor erythroid 2-related factor 2 (Nrf2) (Song et al., 2024), protects NIH-3T3 fibroblasts from H_2_O_2_ damage through the same pathway, and alleviates gastric mucosal injury by boosting glutathione synthesis (Hsuan et al., 2024). Furthermore, AM flower extracts enhance human fibroblast proliferation through cyclin D1 upregulation (Park et al., 2020) and HKC reduces ROS/NO in LPS-activated macrophages and increases lung catalase (CAT)/glutathione peroxidase (GSH-Px) activity in acute lung injury models (Deng et al., 2021). Several studies have revealed that AM exhibits antioxidant activity through scavenging DPPH/ABTS/OH radicals while enhancing antioxidant enzymes (GSH-Px, CAT, SOD, T-AOC) and reducing MDA levels (Qiu et al., 2017; Liu et al., 2021c; Sun et al., 2024). Its total flavonoid fraction TFA activates Nrf2 signaling to upregulate heme oxygenase-1 (HO-1)/NAD(P)H dehydrogenase quinone 1 (NQO1), mitigating D-galactose-induced hepatocyte cytotoxicity (Qiu et al., 2017).

### 5.4 Regulation of intestinal flora

The intestinal microbiota’s pivotal role in IBD pathogenesis is increasingly recognized, with microbial dysbiosis driving immune activation through commensal flora infiltration into lamina propria. Clinical observations reveal reduced microbial diversity in IBD patients, where therapeutic strategies like probiotics and fecal microbiota transplantation (FMT) demonstrate clinical efficacy by restoring ecological balance (Weingarden and Vaughn, 2017; Tao P. et al., 2024; Zhao H. et al., 2024). In DSS-induced colitis models, AM intervention reverses *Firmicutes* depletion and *Bacteroidetes* expansion while enriching beneficial taxa (*Akkermansia muciniphila*, Lachnospiraceae, *Bifidobacterium*) and suppressing pathogens (*Escherichia coli*, *Proteobacteria*) (Zhang W. et al., 2019; Bu et al., 2021). Cohousing experiments demonstrate AM-treated mice attenuate colitis severity in cohabited counterparts (Gao et al., 2022), with fecal microbiota transplantation confirming microbiota-dependent protection via increased *Bacteroides* and *Ruminiclostridium* [sp. cluster 9] in UC recipients (Wu et al., 2021). Notably, AM selectively enhances butyrate-producing Lachnospiraceae to boost short-chain fatty acid (SCFA) production—critical for mucosal integrity and inflammation suppression (Zhang W. et al., 2019) —and directly stimulates A. *muciniphila* proliferation *in vitro* (Bu et al., 2021). In addition, AM polysaccharides enhance mucin production via IL-10-dependent pathways, with bacterial transfer studies confirming *A. muciniphila* as a key mediator of this protective effect (Wang Y. et al., 2024), establishing microbial-metabolic modulation as a viable barrier rehabilitation strategy.

### 5.5 Organ protection

Cerebral ischemia, characterized by insufficient cerebral blood flow leading to oxygen/glucose deficiency, underlies pathologies like cerebral infarction and vascular dementia with high disability/recurrence rates (Chen et al., 2025). AM administration mitigates cerebral edema, attenuates infarct volume, and improves blood-brain barrier integrity while reducing serum biomarkers (LDH, MDA, and PGE2) of neuronal damage in cerebral ischemia-reperfusion injury model (Guo and Chen, 2002; Gao et al., 2003; Wen and Chen, 2006). AM further enhances hippocampal neuron survival by inhibiting intracellular Ca^2+^ overload and lactate dehydrogenase (LDH) release during hypoxia/reoxygenation, while its total flavonoids (TFA) suppress N-methyl-D-aspartate (NMDA) receptor-mediated excitotoxicity via non-competitive receptor desensitization—validated by patch-clamp electrophysiology (Cheng et al., 2006; Wen and Chen, 2017). In post-stroke depression (PSD) models, TFA enhances locomotor activity and improves hemorheological parameters by reducing blood viscosity while increasing erythrocyte deformability. Concurrently, it elevates SOD/GSH-Px activities and reduces levels of MDA, adrenocorticotropic hormone (ACTH), and cortisol in both brain tissue and the hypothalamus (Hao et al., 2007; Hao and Zhou, 2009). These effects are achieved by TFA’s suppression of lipid peroxidation and upregulation of brain-derived neurotrophic factor (BDNF)/cAMP response element-binding protein (CREB) neurotrophic pathways (Liu et al., 2009). In neuronal oxidative stress models, AM ethanol extract directly protects neuronal PC12 cells against oxidative stress through antioxidant enzyme induction, glutathione (GSH) synthesis promotion, DNA repair enhancement, and ROS reduction (Wang S. W. et al., 2022). Critically, AM modulates the gut-brain axis to alleviate depression-associated intestinal barrier damage and microbial dysbiosis, concurrently ameliorated depressive behaviors and DSS-induced colitis (Wang et al., 2021).

AM, traditionally used in Jiangsu and Anhui provinces for jaundice and hepatitis management, exerts hepatoprotection primarily through its total flavonoid component (Liu et al., 2010). In CCl_4_-induced liver injury models, TFA significantly reduces serum markers (ALT, AST, ALP, γ-GT) and hepatic MDA while elevating GSH content, concurrently enhancing antioxidant enzyme activities (SOD, GSH-Px, CAT, GST) and suppressing inflammatory mediators (TNF-α, IL-1β, NO) (Hu et al., 2011; Ai et al., 2013). TFA’s dose-dependent cytoprotection in CCl_4_-exposed hepatocytes, evidenced by improved cell viability and reduced ALT/AST/ALP leakage (Ai et al., 2013). In cholestatic models, TFA upregulates bile transporters (BSEP, MRP2, NTCP) at protein/mRNA levels (Yan et al., 2015). In D-galactose-induced aging models, TFA activates the Nrf2 pathway to boost antioxidant capacity (CAT, T-SOD, GSH-Px, T-AOC) and inhibit peroxidation (Qiu et al., 2017). *In vitro* analyses confirm AM’s ethyl acetate fraction protects HepG2 cells from H_2_O_2_-induced damage through ROS scavenging, membrane stabilization, and apoptosis modulation via Nrf2/HO-1/NQO1 pathway activation and caspase-3/9/Bax regulation (Liu et al., 2021c).

### 5.6 Antiviral, antibacterial, and ulcer repair

AM and its total flavonoid component exhibit broad-spectrum antimicrobial activity against viral and bacterial pathogens. TFA potently inhibits herpes simplex virus types 1/2 (HSV-1 IC_50_ = 1.01 mg/L; HSV-2 IC_50_ = 1.21 mg/L) with high therapeutic indices (>10), indicating selective antiviral action (Jiang et al., 2006). Gao et al. found that AM ethanol extract reduces influenza A viral loads and suppresses pulmonary IL-1β/IL-6/TNF-α via MAPK pathway modulation in murine models (Gao et al., 2022). Against bacterial pathogens, AM demonstrates efficacy against Gram-positive/negative species, particularly *Neisseria gonorrhoeae*, with TFA exhibiting bactericidal activity against *Staphylococcus epidermidis* and *Staphylococcus aureus* (MIC = 3.125 g/L) and fungistatic effects on *Candida albicans* (MIC = 1.562–3.125 g/L) (Zhang et al., 2006). These properties translate to therapeutic efficacy in mucosal infections: TFA accelerates healing of *C. albicans*- or *S. epidermidis*-induced oral ulcers in guinea pigs, and AM flower extract reduces inflammation in *S. aureus*-infected rabbit oral mucosa (Li et al., 2006; Zhang et al., 2006).

### 5.7 Antipyretic and analgesic effects

AM and its total flavonoid component exhibit antipyretic and analgesic properties through distinct biological pathways. In rabbit fever models induced by turpentine oil or *E. coli*, TFA reduced body temperature within 150 minutes—albeit with slower onset than aspirin due to delayed systemic absorption and hepatic metabolism (Fan et al., 2003b). For analgesia, TFA (5–20 mg/kg) significantly inhibited acetic acid-induced writhing responses (42.81%–57.53% reduction) and attenuated potassium chloride-evoked nociception without inducing addictive behaviors even at 280 mg/kg (Fan et al., 2003a). TFA significantly reduced pain responses in rabbits following potassium chloride injections (Fan et al., 2003a). Petroleum ether and methanol extracts of AM also exhibited a dose-dependent inhibition on thermopain (Jain et al., 2011), and AM extracts alleviate burn-induced inflammatory exudation and elevate pain thresholds in scalded models (Song et al., 2024).

## 6 Traditional Chinese medicine formulas containing AM

Currently, the only drug related to *A. manihot* (L.) Medicus (AM) approved for production by the National Medical Products Administration is HKC. Given that there have been a large number of systematic reviews and meta-analyses on the efficacy and safety of HKC (Li et al., 2017; An et al., 2021; Li Y. et al., 2024), we have sorted out the clinical application status of other TCM compounds containing AM (such as Jiahua Tablets, Chuangling Liquid, Yu Kui Qing, etc.), as shown in Table 3. In addition, this section summarizes the traditional prescriptions that contain AM, along with their pharmacological effects (Table 4).

**TABLE 3:** A summary of traditional Chinese medicine compound prescriptions containing AM.

Prescription name	Composition and proportion	Source of the prescription	Clinical application	Ref
Huangkui Capusle	*Abelmoschus manihot* (Huang Shu Kui)	Jiangsu Provincial Hospital of Chinese Medicine	Primary/secondary CKD	Zhang et al. (2014), An et al. (2021)
Jiahua Tablets (甲花片)	*Abelmoschus manihot* (Huang Shu Kui)	Jiangsu Provincial Hospital of Chinese Medicine	Acute and chronic nephritis	Lin et al. (2020a), Wu et al. (2020), Zha et al. (2020)
Chuangling Liquid (疮灵液)	*Abelmoschus manihot* (Huang Shu Kui), *Rheum palmatum* (Da Huang), *Carthamus tinctorius* (Hong Hua), and *Terminalia chebula* (He Zi), and the composition ratio is 1:4:2:2	Jiangsu Provincial Hospital of Traditional Chinese Medicine	Various types of infectious ulcers with significant inflammation	Chen et al., 2017; Pei (2018), Wang et al. (2020)
Huangkui Lianchang Decoction (黄葵敛肠汤)	*Abelmoschus manihot* (Huang Shu Kui), *Euphorbia humifusa Willd* (Di Jin Cao), *Pteris multifida Poir* (Feng Wei Cao), *Lithospermum erythrorhizon Siebold & Zucc* (Zi Cao), *Rubia cordifolia* L. (Qian Cao), and *Rhus chinensis Mill* (Wu Bei Zi), and the composition ratio is 6:6:6:3:3:1	Jiangsu Provincial Hospital of Traditional Chinese Medicine	UC	He et al. (2023), Yang et al. (2023)
Huangkui Siwu Formula (黄葵四物方)	*Abelmoschus manihot* (Huang Shu Kui Hua), *Astragalus mongholicus* (Huang Qi), *Polygonum cuspidatum* (Hu Zhang), and *Curcuma longa* L (Jiang Huang), and the composition ratio is 3.5:5:1.5:1	Nanjing University of Chinese Medicine	CKD	Lu et al. (2020a), Lu et al. (2020b), Lu et al. (2021)
Yu Kui Qing (玉葵清)	*Abelmoschus manihot* (Huang Shu Kui), *Astragalus membranaceus* (Huang Qi), and processed *Polygonum multiflorum* Thunb. (Zhi Shou Wu), and the composition ratio is 30:1.5:1	Not found	Type 2 DN	Chen and Yu (2008), 2009
Qikui Granules (芪葵颗粒)	*Astragalus membranaceus* (Huang Qi), *Abelmoschus manihot* (Huang Shu Kui Hua), and processed *Polygonum multiflorum* (Zhi Shou Wu), and the composition ratio is 3:3:1	Jiangsu Provincial Hospital of Traditional Chinese Medicine	Early to mid-stage DN	Zhang et al. (2021), Wang et al. (2023a)
Huangshu Kuihua paste (黄蜀葵花贴剂)	*Abelmoschus manihot* (Huang Shu Kui)	the First Affiliated Hospital of Anhui Medical University	Managing pain associated with oral ulcers	Han et al. (1997)
Er Huang Ointment (二黄油膏)	*Abelmoschus manihot* (Huang Shu Kui Hua), *Scutellaria baicalensis* (Huang qin), *Phellodendron amurense* (Huang bai), *Gardenia jasminoides* (Zhi zi), and *Ampelopsis japonica* (Bai lian), and the composition ratio is 5:5:5:3:3	Not found	Managing burns and enhancing wound healing	Yang, 2016; Guo and Wang (2017)

**TABLE 4:** Clinical trial of Chinese herbal formula containing AM.

Prescription	Application	Intervention		Subject size	Duration	Outcomes	Conclusion	Ref
		Control group	Treatment group					
Jiahua Tablets	Contrast agent-induced nephropathy	Routine treatment: provided, includingSacubitril/Valsartan (100mg, oral, twice daily), Clopidogrel (75mg, oral, once daily), Atorvastatin (20mg, oral, once daily)	Routine treatment + Jiahua Tablets (4 tablets, PO tid) 3 days preoperatively for 1 week	Control group: 30 cases; Observation group: 30 cases	Preoperative 24h, postoperative 48 h	KIM-1, MCP-1, BUN, SCr, eGFR levels	Jiahua tablet has a certain preventive and treating effect on contrast agent-induced renal injury,we can apply Jiahua tablets during the perioperative period of coronary angiography	Niu et al. (2023)
Jiahua Tablets	Contrast agent-induced nephropathy	Standard treatment (antihypertensives, statins, aspirin) + coronary angiography/PCI; nephrotoxic drugs discontinued 24–48 h prior	Standard treatment + Jiahua Tablets (4 tablets, PO tid) 72 h preoperatively to 72 h postoperatively	Control group: 40 cases; Observation group: 40 cases	Preoperative 24h, postoperative 72 h	SCr, BUN, eGFR, hs-CRP, Cys C, urine NAG/GAL	Jiahua tablet can prevent and treat contrast-induced nephropathy for patients with the perioperative period of coronary intervention	Lin et al. (2020a)
Jiahua Tablets	Postoperative AKI in the patients with PCI	Iodixanol as contrast agent; standard coronary angiography/PCI.	Control treatment + Jiahua Tablets (4 tablets, tid)	Control group: 40 cases; Observation group: 40 cases	Preoperative 72h, postoperative 72 h	Serum SCr, BUN, eGFR, IL-6, IL-8	Jiahua Tablets can be used for the patients with PCI, which can protect and repair AKI by inhibiting the inflammatory response	Lin et al. (2020b)
Jiahua Tablets	Early-stage DN	Diabetic diet + antidiabetic/antihypertensive drugs (ACEIs/ARBs avoided)	Control treatment + Jiahua Tablets (1.8g, tid)	Control group: 30 cases; Observation group: 30 cases	3 months	Serum CRP, TNF-α, Cys C; urinary albumin-to-creatinine ratio (UACR)	The use of Jiahua Tablets based on conventional therapy can effectively reduce serum inflammatory factors levels and improve renal function in patients with diabetic nephropathy	Zha et al. (2020)
Jiahua Tablets	Contrast agent-induced nephropathy	Standard treatment (antihypertensives, statins, aspirin) + coronary angiography/PCI; nephrotoxic drugs discontinued 24–48 h prior	Control treatment + Jiahua Tablets (4 tablets, tid) 72 h preoperatively to 72 h postoperatively for 1 week	Control group: 40 cases; Observation group: 40 cases	Preoperative 72h, postoperative 72 h	SCr, BUN, eGFR, NAG, GAL.	Jiahua tablets can improve the expression of renal injury indexes such as Scr, BUN, NAG and GAL, and exert a better renal protective effect, thereby achieving the purpose of preventing and treating contrast-induced nephropathy	Wu et al. (2020)
Chuangling Liquid	Oral lip ulcer caused by endotracheal intubation	No specific intervention. was implemented	Secure endotracheal tube, clear secretions, clean ulcer with saline; Chuangling Liquid-soaked gauze dressing, bid	20 cases	3–7 days	Wound area, healing rate, healing time	Chuangling Liquid has a good therapeutic effect on lip pressure sores in patients with oral tracheal intubation	Sheng (2014)
Chuangling Liquid	Postoperative wound healing after anal fistula surgery	Debridement + Huangqin Ointment-soaked gauze packing	Debridement + Chuangling Liquid-soaked gauze packing	Huangqin Ointment group: 30 cases; Chuangling Liquid group: 30 cases	2 weeks	Wound area, pain, healing rate, healing time	The wound healing effect of changing the dressing with Chuangling Liquid after surgery for postoperative wound healing after anal fistula surgery is better than Huangqin ointment	Cao et al. (2014)
Chuangling Liquid	Stage III-IV pressure ulcers induced by stroke in the elderly	Routine disinfection/debridement; infected wounds: silver ion/alginate dressings, daily changes	Debridement + heat-sensitive moxibustion + Chuangling Liquid-soaked dressing, once daily	Control group: 15 cases; Observation group: 20 cases	10 days	Granulation growth, wound healing status	The treatment of stage III-IV pressure ulcers in elderly stroke patients with wet compresses using Chuangling Liquid combined with heat-sensitive moxibustion has a remarkable effect	Miao (2013)
Chuangling Liquid	After anal fistula and perianal abscess surgery	Iodophor disinfection + Huangqin Ointment-soaked gauze packing, bid until healing	Iodophor disinfection + Chuangling Liquid-soaked gauze packing, bid until healing	Huangqin Ointment group: 20 cases; Chuangling Liquid group: 20 cases	Not specified	Wound exudate, skin temperature, necrotic sloughing time, healing time	The Chuangling Liquid has a satisfactory effect in reducing exudate from the wound surface, lowering the skin temperature around the wound, accelerating the shedding of necrotic flesh, and promoting healing	Yin et al. (2013)
Chuangling Liquid	Ulcer stage bedsores	Routine disinfection + gentamicin spray + sterile dressing, once/twice daily	Routine disinfection + Chuangling Liquid dressing (daily for exudative wounds); switch to Shengji Yuhong Ointment (bid) when exudate decreases	Control group: 28 cases; Observation group: 28 cases	20 days	Healing rate, granulation growth time, healing timedetect	The clinical effect of the combination Chuangling liquid and Shengji Yuhong ointment used in ulcers of bedsores is significant	Huang and Chen (2012)
Chuangling Liquid	Stage IV pressure ulcer	Infection control + nutrition support; disinfection + ConvaTec dressing (changed when discolored)	Control treatment + Chuangling Liquid moist dressing (weekly changes; extended to 2-3 days with granulation)	Control group: 16 cases; Observation group: 16 cases	4 weeks	Healing rate, granulation time, healing time, dressing change frequency	Five to 7 days after changing the Chuangling Liquid dressing, it can be seen that the necrotic tissue on the wound surface falls off, the black scabs gradually loosen and dissolve, the pus disappears, and the infection is under control	Xu (2010)
Chuangling Liquid	Stage IV pressure ulcer	Infection control + nutrition support; iodophor wet compress + debridement + saline irrigation	Control treatment + Chuangling Liquid moist dressing (weekly changes; extended to 2-3 days with granulation)	Control group: 26 cases; Observation group: 29 cases	30 days	Healing rate, granulation growth time, healing time	The treatment effect of wet compress with Chuangling Liquid combined with iodophor for stage IV pressure ulcers is significantly better than that of wet compress with Chuangling Liquid alone	Xu and Cai (2010)
Chuangling Liquid	Ischemic ulcer	Kangfuxin Solutionappli	Chuangling Liquid-soaked gauze dressing, daily/every other day	Control group: 34 cases; Observation group: 38 cases	3 weeks	Healing rate, pain intensity	The pain-relieving effect of Chuangling Liquid is significantly better than that of other topical liquids, and it is convenient to use with no toxic or side effects at all	Yang (1997)
Chuangling Liquid	Stage II pressure ulcer	Mepilex dressing	Chuangling Liquid + Mepilex dressing	Control group: 30 cases; Observation group: 30 cases	20 days	Wound healing ratedetect	The combination of Chuangling Liquid and Mepilex has a definite therapeutic effect on stage II pressure ulcers, which can shorten the healing time of the ulcer surface and increase the healing rate	Teng and Ji (2016)
Chuangling Liquid	Chronic lower limb ulcers	Vaseline-soaked gauze, dressing changes every other day	Two subgroups: Chuangling Liquid- soaked gauze or Chuangling Liquid- loaded collagen, changes every other day	Chuangling Liquid collagen group: 20 cases; Chuangling Liquid group: 20 cases; Vaseline group: 20 cases	14 days	Healing rate, inflammation/ granulation scores, infection rate	After the Chuangling Liquid is loaded into the collagen sponge, it helps to enhance the therapeutic effect of the Chuangling Liquid in regulating wound inflammation and the collagen sponge in promoting granulation tissue growth, further promoting the healing of chronic wounds	Wang et al. (2016)
Chuangling Liquid	Postoperative mixed hemorrhoids	Vaseline-soaked gauze strips for dressing changes	Chuangling Liquid-soaked gauze + photon therapy device irradiation	Control group: 20 cases; Observation group: 20 cases	7 days	Edema, exudate, pain, healing time	Chuangling solution and photon therapeutic instrument combined with high-quality effective nursing measures for postoperative mixed hemorrhoids has exactly curative effect	Li (2017)
Chuangling Liquid	After anal fistula and perianal abscess surgery	Huangqin Ointment-coated gauze strips for dressing changes	Chuangling Liquid-soaked gauze strips for dressing changes	Control group: 20 cases; Observation group: 20 cases	11 days	Exudate, skin temperature, necrotic sloughing time, healing time	The anal fistula and perianal abscess after surgery using Chuangling lotion for dressing exchange can reduce the wound-surface exudate and inflammation reaction and promote wound-surface healing	Xu (2017)
Chuangling Liquid	Diabetic foot	Glucose/blood pressure/lipid control + antibiotics + microcirculation improvement + debridement + saline irrigation	Control treatment + herbal fumigation + Chuangling Liquid moist dressing, bid for 4 weeks (total 16 weeks)	Control group: 30 cases; Observation group: 30 cases	16 weeks	Wound healing status, hospitalization costs	Chuangling Liquid combined with herbal fumigation and washing can promote the healing of diabetic foot	Pei (2018)
Chuangling Liquid	Diabetic foot	Photon therapy device irradiation, bid for 15–20min	Photon therapy + Chuangling Liquid moist dressing, daily changes	Control group: 60 cases; Observation group: 60 cases	2 weeks	Exudate score, dressing change pain score, healing time, granulation time	In observation group, significant effects were achieved in reducing the wound exudate score, lowering the pain score during dressing change, shortening the healing time and granulation tissue growth time	Liu (2019)
Chuangling Liquid	Puncture site infection after PICC placement	Diluted iodophor wet compress on puncture site	Chuangling Liquid wet compress on puncture site	Control group: 18 cases; Observation group: 18 cases	5 days	Healing effect, healing time, dressing change frequency, cost	Chuangling solution wet-compressing is effective in the treatment of puncture site infection after PICC placement,and associated with shorter healing time and less frequency and cost of dressing change compared with povidone-iodine-diluent wet-compressing	Wang et al. (2020)
Chuangling Liquid	Plasma cell mastitis (PCM)	Iodophor disinfection + pus drainage + granulation scraping (if any) + saline irrigation + saline gauze drainage	Control treatment + Chuangling Liquid irrigation + Chuangling Liquid-soaked gauze drainage, dressing changes twice weekly	Control group: 20 cases; Observation group: 20 cases	4 weeks	Tumor size, patient pain score, and levels of IL-1β, IL-2, IL-6, IFN-γ, and TNF-α were measured	External use of Chuangling Liquid inhibited local inflammation and reduced local mass of PCM patients.	Xue et al. (2020)
Chuangling Liquid	Non-lactating mastitis	After abscess incision and drainage, the wound and surrounding area were routinely disinfected with iodophor. The wound and secretions were rinsed with normal saline, and regular gauze strips or Vaseline gauze strips were used for drainage	After abscess incision and drainage, the wound and surrounding area were routinely disinfected with iodophor. The wound and secretions were rinsed with Chuangling Liquid, and Chuangling Liquid-soaked gauze strips were used for drainage	Control group: 33 cases; Observation group: 31 cases	4 weeks	Wound symptom score and levels of wound tissue inflammatory markers (TNF-α, IFN-γ, IL-1β, IL-2, IL-6) were measured	The external application of Chuangling Liquid can effectively improve the local symptoms and improve the curative effect by reducing the inflammation of the wound tissue in the treatment of non-lactating mastitis patients with local ulceration	Ye et al. (2021)
Huangkui Lianchang Decoction	UC	Mesalazine enteric-coated tablets, 1 g PO TID.	Huangkui Lianchang Decoction administered via retention enema	Control group: 60 cases; Observation group: 60 cases	12 weeks	Baron endoscopy score, Mayo score, and scores of main UC symptoms (diarrhea, abdominal pain, mucus, rectal bleeding, tenesmus) were evaluated	The retention enema of Huangkui Lianchang Decoction has a good therapeutic effect on UC with damp-heat internal accumulation syndrome. It can inhibit the inflammatory response of the intestinal mucosa in patients, reduce mucosal damage, and has good safety	Yang et al. (2023)
Yu Kui Qing	DN	Yu Kui Qing placebo (dextrin granules), 1 packet PO BID daily	Yu Kui Qing granules, 1 packet PO BID.	Control group: 30 cases; Observation group: 31 cases	12 months	Urinary mAlb/Cr levels were measured before and after treatment. Symptom scores were observed, quantifying symptoms such as fatigue, lower limb edema, back pain, frequent urination, increased nocturia, and dry mouth	Yukuiqing has the effect of reducing urinary microalbumin and can improve the symptoms of fatigue, weakness and lower back pain in patients with early type 2 DN.	Chen and Yu (2008)
Qikui Granules	DN	Standard hypoglycemic (excluding thiazolidinediones) and antihypertensive (ARB as first-line) treatment, with FBG 4.4–7.0 mmol/L, PBG 4.4–10.0 mmol/L, BP < 130/85 mmHg	Control group treatment + Qikui Granules (1 bag PO TID, 150–200 mL warm water 1 h post-meal)	Control: 32 cases; Treatment: 31 cases	12 weeks	UACR, UAER, inflammatory factors (IL-6, TNF-α, TGF-β1, MCP-1)	Qikui Granules can significantly reduce urinary protein in patients with type 2 DN. Its mechanism of action may be to exert a protective effect on the kidneys by improving the inflammatory state of the glomerulus	Xie et al. (2017a)
Qikui Granules	DN	Standard hypoglycemic (conventional agents/insulin) and antihypertensive (excluding ACEI/ARB) treatment, maintaining normal BP.	Control group treatment + Qikui Granules (1 bag PO TID)	Control: 50 cases; Treatment: 52 cases	6 months	BP, BG, BUN, CR, 24hUTP, urinary CTGF, serum sICAM-1	Qikui Granules can significantly reduce urinary protein in patients with type 2 DN. Its mechanism of action may be to exert a protective effect on the kidneys by improving the inflammatory state of the glomerulus	Zhang et al. (2021)
Qikui Granules	DN in elderly patients	Dulaglutide (1.5 mg SC QW)	Dulaglutide (1.5 mg SC QW) + Qikui Granules (10 g PO TID)	Control: 40 cases; Treatment: 40 cases	12 weeks	BG (FPG, HbA1c), BP (SBP, DBP), lipids (TC, TG, LDL-C, HDL-C), renal indicators (SCr, UACR, 24hUTP)	The combination of Qikui Granules and Dulagtide can significantly reduce the levels of Scr and urine protein in elderly patients with DKD in the clinical stage, and has a good protective effect on the kidneys	Wang et al. (2023a)
Huangshu Kuihua paste	Oral mucosal ulcer	Compound borax gargle + Xileisan (blown on lesion 5 times/d)	Compound borax gargle + Huangshu Kuihua Paste (covered lesion, held 15min before swallowing, 3 times/d)	Control: 36 cases; Treatment: 82 cases	5 days	Pain, eating status, ulcer healing	Compared with the Xileisan group, the total effective rate of the Huangshu Kuihua paste was significantly higher	Han et al. (1997)
Er Huang Ointment	Superficial second-degree burns	SD-Ag cream (QD)	Er Huang Ointment + rhGM-CSF gel (BID-TID)	Control: 49 cases; Treatment: 49 cases	2 weeks	Wound healing rate, scarring, healing time, pain, adverse reactions	The combination of Er Huang Ointment and rhGM-CSF gel can effectively promote wound healing in patients with superficial second-degree burns and reduce scarring	Guo and Wang (2017)
Er Huang Ointment	Superficial second-degree burns	Vaseline (QD)	Er Huang Ointment (QD)	Control: 45 cases; Treatment: 45 cases	1 week	Healing time, healing rate, visual analogue scale, skin irritation, appearance satisfaction	The clinical efficacy of Er Huang Ointment in treating superficial second-degree burns is remarkable. It can accelerate the wound healing time, relieve pain, avoid severe irritation to the skin, and has a relatively high safety level	Yang (2016)

### 6.1 Jiahua tablets

Jiahua Tablets are semi-extracted TCM tablets made from the raw powder and extracts of AM flowers, containing 10% starch slurry and an appropriate amount of ethanol. These tablets have been clinically used for over a decade and have been demonstrated to display significant therapeutic efficacy (Lin et al., 2020a; Zha et al., 2020). Both HKC and Jiahua Tablets are derived from the single medicinal ingredient of AM flowers. They share properties that clear heat, promote diuresis, support kidney function, and eliminate toxins, primarily addressing acute and chronic nephritis (Zhang and Zhou, 2012). According to the *Chinese Pharmacopoeia*, the recommended dosage of AM flowers is 10–30 g/day, with both HKC and Jiahua Tablets containing a daily dosage of 30 g of AM flowers.

In addition to conventional early DN treatments, Jiahua Tablets can reduce serum inflammatory factors such as C-reactive protein (CRP) and TNF-α. It also lowers levels of Cystatin C (Cys-C) and urinary albumin-to-creatinine ratio (UACR), thereby improving kidney function (Cha et al., 2020). In patients undergoing coronary intervention, those treated with Jiahua Tablets exhibited significantly lower levels of serum creatinine (SCr), BUN, Cys-C, IL-6, IL-8, high-sensitivity C-reactive protein (hs-CRP), urinary N-acetyl-β-D-glucosaminidase (NAG), and urinary β-galactosidase (GAL) 72 h post-operation compared to the control group. Additionally, the estimated glomerular filtration rate (eGFR) was significantly higher in the treatment group than in the control group, indicating that Jiahua Tablets can effectively prevent contrast-induced nephropathy. The underlying mechanism likely involves inhibiting inflammatory responses and protecting renal tubules, thereby repairing kidney damage and preserving renal function (Wu et al., 2020).

### 6.2 Chuangling liquid

Chuangling Liquid was developed by the Jiangsu Provincial Hospital of TCM in the 1980s. Its formulation including *A. manihot* (Huang Shu Kui), *Rheum palmatum* (Da Huang), *Carthamus tinctorius* (Hong Hua), and *Terminalia chebula* (He Zi) (Yang, 1997; Xu, 2010; Huang and Chen, 2012; Miao, 2013; Yin et al., 2013; Cao et al., 2014; Sheng, 2014; Teng and Ji, 2016; Wang et al., 2016; Li, 2017; Liu, 2019; Wang et al., 2020; Xue et al., 2020; Ye et al., 2021). The primary active components of Chuangling Liquid include gallic acid, hyperoside, isoquercitrin, myricetin, quercetin, rhein, emodin, chrysophanol, and physcion (Shu et al., 2016). This preparation promotes blood circulation and removes blood stasis, effectively treating various types of infectious ulcers with significant inflammation (Chen et al., 2017).

Chuangling Liquid has demonstrated a range of therapeutic properties in various experimental and clinical settings. Zhang et al. showed that it can counteract acute exudative inflammation induced by croton oil in mice and inhibit granulation tissue proliferation in cotton pellet granuloma models (Zhang et al., 1997). In rabbit ulcer models, Chuangling Liquid promotes early wound healing processes such as epithelial tissue proliferation, granulation formation, and accessory regeneration, while inhibiting collagen fiber scar formation, leading to faster healing with less scarring (Zhang et al., 1997). Additionally, it exhibits strong antibacterial activity against *S. aureus*, methicillin-resistant *S. aureus* (MRSA), and *Proteus species*, being particularly effective at eliminating MRSA from infected wounds and promoting recovery (Liu et al., 2018). Moreover, Chuangling Liquid also inhibits thrombosis and platelet aggregation, thereby improving blood circulation and alleviating blood stasis (Chen et al., 2017). Clinically, it has been proven effective in treating postoperative infected wounds, chronic ulcers, diabetic foot ulcers, venous ulcers of the lower limbs, and anal fistulas (Hu, 2016; Li, 2016; Xu, 2017). Furthermore, modifying the formulation process and incorporating collagen into Chuangling Liquid enhances its anti-inflammatory effects, wound healing, angiogenesis, and overall recovery (Zhao and Zhu, 2013). Recent studies have also found that Chuangling Liquid promotes the proliferation of immortalized melanocytes in mice and activates tyrosinase activity, enhancing melanin synthesis. This suggests potential applications in treating pigmentary disorders such as vitiligo (Zhou et al., 2019).

### 6.3 Huangkui Lianchang decoction

Huangkui Lianchang Decoction (HLD) is a traditional Chinese medicinal formulation used to treat UC. It consists of six key ingredients: the primary herb is *A. manihot* (Huang Shu Kui), combined with *Euphorbia humifusa Willd* (Di Jin Cao), *Pteris multifida Poir* (Feng Wei Cao), *Lithospermum erythrorhizon Siebold* & *Zucc* (Zi Cao), *Rubia cordifolia* L. (Qian Cao), and *Rhus chinensis Mill* (Wu Bei Zi) (He et al., 2019; Yang et al., 2023). HLD is rich in flavonoids, including rutin, isoquercitrin, gossypetin, and quercetin (Cheng et al., 2022). The proposed functions of HLD are to clear heat and dampness, activate blood circulation, protect the intestines, and detoxify the body. These properties make it particularly beneficial for managing the symptoms associated with UC.

HLD has demonstrated significant therapeutic effects on DSS-induced UC in mouse models. Studies indicate that HLD exerts anti-inflammatory effects by modulating key signaling pathways, including NF-κB, IL-6/signal transducer and activator of transcription 3 (STAT3), and the IL-23/IL-17 inflammatory axis (He et al., 2019; He et al., 2020; Cheng et al., 2022). *In vitro* experiments with drug serum containing HLD (HLD-DS) showed that it reduces inflammatory cytokine levels while increasing IL-10 levels in NCM460 cells. HLD-DS also decreased the expression of p-NF-κB p65, LC3II/I, and Beclin 1, suggesting that HLD alleviates colitis by inhibiting the NF-κB pathway and autophagy (Cheng et al., 2022). Clinically, HLD is used for treating mild to moderate E1 and E2 type UC via enema alone. In contrast, severe or E3-type UC is treated with HLD enemas and oral medications (He et al., 2019). Clinical studies have shown that modified HLD, when used alongside corticosteroids, can effectively improve gut microbiota and exhibit significant anti-inflammatory effects, aiding in managing IBD (He et al., 2023). Additionally, the retention enema method of HLD has been found to inhibit the intestinal mucosal inflammatory response in UC patients and reduce mucosal damage (Yang et al., 2023).

### 6.4 Huangkui Siwu Formula

Huangkui Siwu Formula (HKSWF) is a traditional Chinese medicinal formulation comprised of four key herbs: *A. manihot* (Huang Shu Kui), *Astragalus mongholicus* (Huang Qi), *Polygonum cuspidatum* (Hu Zhang), and *Curcuma longa* L (Jiang Huang) (Lu T. et al., 2020). The formula contains eleven identified components, including polydatin, hyperoside, isoquercitrin, hibifolin, myricetin, resveratrol, quercetin, didemethoxycurcumin, demethoxycurcumin, curcumin, and emodin (Lu T. et al., 2020). The herbal combination in HKSWF clears heat, promotes blood circulation, facilitates urination, and reduces swelling, effectively addressing the symptoms of CKD.

Research on HKSWF has shown promising therapeutic effects in kidney health, particularly in an anti-Thy-1 nephritis model. One study indicates that HKSWF can alleviate glomerular injury by attenuating pyruvate dehydrogenase activity, contributing to its protective effects on renal function (Lu T. et al., 2020). Further investigations by Lu et al. revealed that HKSWF does not alter the expression of organic anion transporters in the kidneys or the transport of p-cresyl sulfate (PCS) from blood to kidneys. Instead, it regulates the synthesis pathway of PCS within host cells, inhibiting its endogenous production. This action helps reduce the accumulation of uremic toxins and slows the progression of CKD (Lu et al., 2021). Moreover, HKSWF modulates uremic toxin metabolism pathways within the gut microbiota, inhibiting the formation of uremic toxin precursors at various stages. This modulation alleviates the symptoms associated with uremic toxin accumulation and further delays CKD progression, highlighting the multifaceted benefits of HKSWF for managing kidney health (Lu J. et al., 2020).

### 6.5 Yu Kui Qing

Yu Kui Qing (YKQ) is composed of *A. manihot* (Huang Shu Kui), *Astragalus membranaceus* (Huang Qi), and *Polygonum multiflorum* Thunb. (Zhi Shou Wu) (30:1.5:1) (Chen and Yu, 2008; Chen and Yu, 2009). YKQ clears heat, detoxifies, promotes diuresis, tonifies Qi, strengthens the spleen, activates blood circulation, and nourishes Yin and kidneys. Modern pharmacological studies have demonstrated that YKQ can reduce the expression and chemotactic effects of chemokines in human renal mesangial cells (HRMC) induced by advanced glycation end-products bound to bovine serum albumin (AGE-BSA). Additionally, YKQ intervenes in the expression of connective tissue growth factor (CTGF) mRNA and protein in HRMC, which may contribute to reducing renal inflammation associated with DN (Yang et al., 2010; Sun et al., 2014). Clinical studies have shown that YKQ effectively treats microalbuminuria in patients with type 2 DN, reduces urinary microalbumin levels, and improves symptoms (such as fatigue and lower back pain) in early-stage disease. These findings underscore the potential of YKQ as a valuable therapeutic option for managing diabetes-related complications (Chen and Yu, 2009).

### 6.6 Qikui Granules

Qikui Granules is composed with *A. membranaceus* (Huang Qi), *A. manihot* (Huang Shu Kui), and processed *P. multiflorum* (Zhi Shou Wu) (Wang L. et al., 2023). Specifically, they help to benefit Qi, nourish Yin, and clear heat. Clinically, Qikui Granules have been utilized to treat early to mid-stage DN (Yang et al., 2016).

The study by Lou et al. shows that Qikui Granules provide renal protection in DN by lowering blood glucose, reducing inflammatory markers like IL-6, monocyte chemoattractant protein-1 (MCP-1), and TGF-β1, and inhibiting the p38 MAPK signaling pathway (Yang et al., 2016; Shao et al., 2017). In addition to renal benefits, Qikui Granules were observed to improve bone density, likely linked to improved blood glucose regulation (Lou et al., 2022). *In vitro* experiments revealed that these granules enhance the proliferation of mesenchymal stem cells (MSCs) and promote their osteogenic differentiation while simultaneously reducing their potential for adipogenic differentiation (Lou et al., 2022). Clinically, Qikui Granules have been shown to reduce microalbuminuria in patients with early DN. It slows disease progression and alleviates symptoms such as fatigue, weakness, lower back and knee soreness, and facial and limb edema (Yan et al., 2018). Observations indicate a marked reduction in urine protein levels, UACR, urinary albumin excretion rate (UAER), and urinary MCP-1/creatinine (uMCP-1/Cr) levels in patients with early DN (Yan et al., 2018). The underlying mechanisms of action for Qikui Granules may involve inhibiting CTGF expression and soluble intercellular adhesion molecule-1 (sICAM-1) in urine and serum (Xie et al., 2017b; Zhang et al., 2021). Overall, the findings suggest that Qikui Granules could be a valuable therapeutic approach for managing complications associated with diabetes, particularly DN, by addressing metabolic and inflammatory pathways.

### 6.7 Huangshu Kuihua paste

The Huangshu Kuihua paste, which combines 0.3–10 parts of *A. manihot* (Huang Shu Kui) with 0.1–10 parts of propolis extract and 0.1-5 parts of borneol, is an innovative formulation widely used at the First Affiliated Hospital of Anhui Medical University (Han and Situ, 1997; Chen et al., 2017). Its strong analgesic properties make it particularly effective for managing pain associated with oral ulcers. Notably, the formulation is characterized by minimal tissue irritation and does not cause local numbness or discomfort, which enhances patient comfort during treatment. Moreover, the paste exhibits excellent adhesion to mucous membranes, facilitating prolonged contact and promoting the healing process of oral ulcers without any toxic side effects. In clinical trials involving 82 patients with oral ulcers, the formulation achieved an impressive cure rate of 97.5%, underscoring its efficacy and safety in treating this condition (Han et al., 1997). These findings make Huangshu Kuihua paste a valuable option in managing oral ulcer pain and healing.

### 6.8 Er Huang ointment

Er Huang Ointment, formulated with *A. manihot* (Huang Shu Kui), *Scutellaria baicalensis* (Huang qin), *Phellodendron amurense* (Huang bai), *Gardenia jasminoides* (Zhi zi), and *Ampelopsis japonica* (Bai lian) (5:5:5:3:3), has garnered attention for its therapeutic effects on burns and wounds (Yang, 2016). Clinical studies have affirmed that the overall efficacy of Er Huang Ointment in treating burns and scalds and reducing wound healing time is comparable to that of Moist Burn Ointment (Yang et al., 2016; Guo and Wang, 2017). Furthermore, Er Huang Ointment has notable antibacterial activity against common pathogens such as *S. aureus*, *Pseudomonas aeruginosa*, and *E. coli*, suggesting its potential in preventing infections associated with burns and wounds (Li, 1993). These findings position Er Huang Ointment as a promising clinical practice option for managing burns and enhancing wound healing.

## 7 Research challenges and future perspectives

The traditional Chinese medicinal herb AM features pale yellow flowers with a delicate fragrance and holds significant ornamental, medicinal, and edible value. Modernizing AM faces two core challenges: degree of development and utilization, and gaps in translation from laboratory to clinic. *Jiangsu Suzhong Pharmaceutical Co., Ltd.* in Taizhou (Jiangsu, China) has successfully developed and commercially produced HKC—a Category III new TCM—after years of research. These capsules are widely used clinically to treat conditions such as nephritis and rheumatoid arthritis, serving as an adjunctive therapy for glomerulonephritis and demonstrating considerable value in preventing gadolinium-based contrast agent-induced nephrogenic systemic fibrosis. Utilizing AM flowers as the primary raw material, the proven efficacy and substantial market potential of these capsules have driven high demand, consequently stimulating the flourishing cultivation industry across Taizhou, Jiangsu, surrounding regions, and nationwide. However, other AM parts—including leaves, roots, stems, and seeds—are typically discarded, resulting in resource wastage and environmental pollution. Literatures indicate that the entire AM plant possesses medicinal properties, suggesting that non-capsule components also hold therapeutic value. To enable more scientific, comprehensive, and rational utilization of this medicinal resource, intensified research on its other bioactive parts is essential. This will maximize economic and social benefits while accelerating the development of the AM industry.

Transitioning to evidence-based medicine is hampered by significant clinical evidence gaps, including small randomized controlled trials with fewer than 300 cases, short follow-up periods of 6 months or less, reliance on surrogate endpoints like proteinuria instead of hard endpoints such as end-stage renal disease incidence, and insufficient long-term safety data on aspects like reproductive toxicity. Currently, only HKC has gained broad clinical acceptance, primarily for treating nephropathy. This is due to its robust clinical data, characterized by rigorous record-keeping, standardized patient grouping, sufficient sample sizes, and sustained post-treatment follow-up. Other AM-containing TCM formulations suffer from significant clinical evidence gaps: their studies often involve small, non-systematic patient cohorts and lack post-administration observation. This evidence deficit severely limits the broader clinical adoption and justification for using AM. Therefore, building upon the systematically compiled data on AM-containing TCM formulations presented in this study, it is recommended to prioritize 1-2 promising prescriptions for development. Conducting standardized clinical research on these selected formulations is crucial. This focused approach will enhance the understanding of AM’s therapeutic potential and ultimately expand its clinical applications.

## 8 Conclusion

Based on the current body of evidence derived from both basic research and clinical practice, AM has demonstrated significant therapeutic potential. Its pharmacological activities, including the improvement of liver and kidney functions, regulation of material metabolism, and anti-fibrotic effects, have been scientifically validated, providing a rationale for its application in conditions such as CKD and IBD (Miao et al., 2024a; Wang Y. et al., 2024; Zhao H. et al., 2024). However, the predominant clinical application remains limited to the single-component preparation HKC (Geng et al., 2023), underscoring the need to broaden the development and utilization of AM-derived formulations.

Notwithstanding this promise, current research faces notable limitations that must be addressed in future studies. These include a narrow focus on the flowers and total flavonoids, neglecting other plant parts and individual bioactive compounds; the use of animal models that may not accurately replicate human diseases; insufficient mechanistic insights at cellular and molecular levels; and a lack of integrated pharmacokinetic-pharmacodynamic (PK-PD) studies to represent the whole herb’s *in vivo* behavior. Consequently, future efforts should prioritize expanding phytochemical investigations, pinpointing specific bioactive compounds and their mechanisms, establishing integrated PK-PD models, and developing improved formulations through rigorous clinical trials. Overcoming these challenges via a multidisciplinary approach is essential to fully realize the clinical potential of AM.

## Glossary

ACEI: angiotensin-converting enzyme inhibitor
ACTH: adrenocorticotropic hormone
AGEs: advanced glycation end products
AGE-BSA: advanced glycation end-products bound to bovine serum albumin
AKI: acute kidney injury
AKT: protein kinase B
ALI: acute lung injury
ALP: alkaline phosphatase
ALT: alanine aminotransferase
AM: *Abelmoschus manihot* (L.) Medicus
AMPK: adenosine monophosphate-activated protein kinase
ARB: angiotensin II receptor blocker
ASC: apoptosis-associated speck-like protein containing caspases recruitment domain
AST: aspartate aminotransferase
Bax: Bcl-2-associated x protein
Bcl-2: B-cell lymphoma-2
BDNF: brain-derived neurotrophic factor
BECN1: beclin 1
BG: blood glucose
bid: bis in die
BP: blood pressure
BSEP: bile salt export pump
BUN: blood urea nitrogen
**[Ca** ^**2+**^ **]**_**i**_: calcium concentrations
CAM: chick chorioallantoic membrane
CAT: catalase
CCL: C-C Motif Chemokine Ligand
C/EBPα: CCAAT/enhancer-binding protein alpha
CKD: chronic kidney disease
COX-2: cyclooxygenase-2
CTGF: connective tissue growth factor
Cys C: cystatin C
CREB: cAMP response element-binding protein
CRP: C-reactive protein
DAI: disease activity index
DBP: diastolic blood pressure
DN: diabetic nephropathy
DKD: diabetic kidney disease
DPPH: 2,2-diphenyl-1-picrylhydrazyl
DSS: dextran sodium sulfate
EPCs: endothelial progenitor cells
ERK1/2: extracellular signal-regulated kinase 1/2
EMT: epithelial-mesenchymal transition
eGFR: estimated glomerular filtration rate
FBG: fasting blood glucose
FPG: fasting plasma glucose
FST: forced swimming test
GAL: β-galactosidase
GLUT-4: glucose transporter type 4
GSH: glutathione
GSH-Px: glutathione peroxidase
GST: glutathione S-transferase
HbA1c: glycated hemoglobin A1c
HG: high glucose
HIF-1a: hypoxia-inducible factors 1a
HKC: Huangkui Capsule
HLD: Huangkui Lianchang decoction
HLD-DS: drug serum containing HLD
HO-1: heme oxygenase-1
HRMC: human renal mesangial cells
HKSWF: Huangkui Siwu Formula
HUVECs: human umbilical vein endothelial cells
hs-CRP: high-sensitivity C-reactive protein
ICAM-1: intercellular adhesion molecule-1
IFN-γ: interferon-gamma
*i.g*: intragastric administration
IgA nephropathy: immunoglobulin a nephropathy
IL-1β: interleukin-1β
iNOS: inducible nitric oxide synthase
IκBα: nhibitor of nuclear factor kappa b alpha
**I**_**NMDA**_: N-methyl-D-aspartate receptor-activated current
JAK2: janus kinase 2
LC3: microtubule - associated protein light chain 3
LDL-C: low-density lipoprotein cholesterol
LDH: lactate dehydrogenase
LPS: lipopolysaccharide
MAdCAM-1: mucosal addressin cell adhesion molecule-1
mAlb/Cr: microalbumin/creatinine ratio
MAPK: mitogen-activated protein kinase
MCP-1: monocyte chemoattractant protein-1
MDA: malondialdehyde
MPO: myeloperoxidase
MRSA: methicillin-resistant *Staphylococcus aureus*
MRP2: multidrug resistance-associated protein 2
MYD88: myeloid differentiation primary response 88
NADPH: nicotinamide adenine dinucleotide phosphate
NAG: N-acetyl-β-D-glucosaminidase
NF-κB: nuclear factor kappa B
NLRP3: nod-like receptor protein 3
NMDA: N-methyl-D-aspartate
Nrf2: nuclear factor erythroid 2-related factor 2
NO: nitric oxide
NOX4: nicotinamide adenine dinucleotide phosphate oxidases 4
NOS: nitric oxide synthase
NS: nephrotic syndrome
NTCP: sodium taurocholate cotransporting polypeptide
ox-LDL: oxidized low-density lipoprotein
PBG: postprandial blood glucose
PCI: percutaneous coronary intervention
PCS: p-cresyl sulfate
PGE2: prostaglandin E2
PICC: peripherally inserted central catheter
PI3K: phosphoinositide 3-kinase
po: peros
PPARγ: peroxisome proliferator-activated receptor gamma
PSD: post-stroke depression
PTZ: pentylenetetrazol
qd: quaque die
qw: quaque week
rhGM-CSF: granulocyte-macrophage colony-stimulating factor
ROS: reactive oxygen species
SBP: systolic blood pressure
SC: subcutaneous
SCFAs: short-chain fatty acids
SCr: serum creatinine
sICAM-1: soluble intercellular adhesion molecule-1
SIRT1: sirtuin 1
Slc: solute carrier family
Smad3: smad family member 3
STAT3: signal transducer and activator of transcription 3
SOCS1: suppressor of cytokine signaling 1
SOD: superoxide dismutase
KDR: kinase insert domain receptor
KIM-1: kidney injury molecule-1
TACE: tumor necrosis factor-α converting enzyme
T-AOC: total antioxidant capacity
TBA: total bile acid
TC: total cholesterol
TCM: traditional Chinese medicine
TFA: total flavones of *Abelmoschus manihot*
TG: triglyceride
TGF-β: transforming growth factor beta
Th17: T helper 17 cell
TNBS: 2,4,6-trinitrobenzenesulfonic acid solution
TNF-α: tumor necrosis factor-alpha
tid: ter in die
TLR4: toll-like receptor 4
Treg: regulatory T cell
UACR: urinary albumin-to-creatinine ratio
UAER: urinary albumin excretion rate
UC: ulcerative colitis
UTP: urinary total protein
VEGF-A: vascular endothelial growth factor-A
VEGFR2: vascular endothelial growth factor receptor-2
VCAM-1: vascular cell adhesion molecule-1
YKQ: Yu Kui Qing
ZO-1: zonula occludens-1
